# Biomass partitioning of plants under soil pollution stress

**DOI:** 10.1038/s42003-022-03307-x

**Published:** 2022-04-19

**Authors:** Florian Delerue, Mathieu Scattolin, Olivier Atteia, Gregory J. V. Cohen, Michel Franceschi, Michel Mench

**Affiliations:** 1 Univ. Bordeaux, CNRS, Bordeaux INP, EPOC, UMR 5805, F-33600 Pessac, France; 2 Mines Saint-Etienne, Univ Lyon, Univ Jean Moulin, Univ Lumière, Univ Jean Monnet, ENTPE, INSA Lyon, ENS Lyon, CNRS, UMR 5600 EVS, Centre SPIN, Département PEG, F-42023 Saint-Etienne, France; 3 Univ. Bordeaux, INRAE, BIOGECO, F-33615 Pessac, France

**Keywords:** Ecophysiology, Plant ecology, Plant stress responses

## Abstract

Polluted sites are ubiquitous worldwide but how plant partition their biomass between different organs in this context is unclear. Here, we identified three possible drivers of biomass partitioning in our controlled study along pollution gradients: plant size reduction (pollution effect) combined with allometric scaling between organs; early deficit in root surfaces (pollution effect) inducing a decreased water uptake; increased biomass allocation to roots to compensate for lower soil resource acquisition consistent with the optimal partitioning theory (plant response). A complementary meta-analysis showed variation in biomass partitioning across published studies, with grass and woody species having distinct modifications of their root: shoot ratio. However, the modelling of biomass partitioning drivers showed that single harvest experiments performed in previous studies prevent identifying the main drivers at stake. The proposed distinction between pollution effects and plant response will help to improve our knowledge of plant allocation strategies in the context of pollution.

## Abstract (web summary)

An empirical study with different levels of soil pollution and a meta-analysis provide insight into the drivers of plant biomass partitioning under soil pollution stress.

## Introduction

Soil pollution has adverse effects on ecosystem functioning and creates risks for the environment and human health. It is a major global environmental issue. The US Environmental Protection Agency tracks nearly 9 million ha of possibly contaminated land^1^ and in China, 19% of agricultural soils show contamination exceeding the levels of environmental quality standards^2^. In Europe, 2.5 million sites are potentially contaminated and 350,000 of them require remediation^3^. Metal(loid)s and polycyclic aromatic hydrocarbons (PAH) contribute considerably to this contamination^3,4^. Considering this major issue, a large body of scientific literature has investigated the ecotoxic effects of pollutants on plant species and in-situ phytomanagement options. The accumulated knowledge shows that toxic effects depend on the subtle interplay between plant species and/or genotype, contaminant type, soil matrix, and any interaction with soil organisms. Consequently, designing management options often requires demanding characterisation of physical and chemical polluted soil properties and the evaluation of soil ecotoxicity on several selected plants/genotypes or other organisms. Meanwhile, remediation of the numerous polluted sites worldwide remains challenging. A simpler and more unified framework to study ecosystem functioning in polluted contexts, especially in terrestrial systems^5^ should help to meet this challenge.

Pollution affects plant growth in complex ways. For instance, in addition to direct toxic effects on plant metabolism, the development of plant parts responsible for resource capture (mainly light, water, and nutrients) can be impacted, creating an indirect decrease in resources available for growth. Plants exposed to various contaminants, e.g. PAH^6,7^, or metal(loid)s^8^, exhibit thicker roots, reduction of root elongation, and thickened epidermis, cortex or endoderm. Consequently, the root absorptive surface and soil resource uptake decrease^9^. A more unified framework for the study of plant response in polluted areas has to encompass these direct and indirect constraints for plant growth.

Despite the complexity of many environmental gradients, ecologists have provided a set of consistent theories based on general natural laws to overcome this complexity. One of them is the “Optimal Partitioning Theory (OPT)”^10–12^ which focuses on plant response to variation in resource availability. To maintain the homeostasis of the different resources necessary for growth, biomass can be allocated in priority to the construction of the organs responsible for the capture of the most limiting resource. It results in a higher mass fraction devoted to shoots and leaves in shaded environments, and a higher mass fraction devoted to roots in nutrient or water-limited ones (see ref. ^13^ for a detailed review). The way plants partition biomass is one of the cornerstones of plant growth strategies. To reach a general understanding of plant response to soil pollution, this study focused on the drivers of biomass partitioning along soil pollution gradients, as an expression of both the direct toxic impact on plant growth and plant response to the indirect decrease of resource availability for growth.

There are four main causes, all of which are related to changes in resource availability, implying that changes in biomass partitioning could be involved in plant response to soil pollution. Here, we considered gradients encompassing situations ranging from low to high phytotoxic effects. Firstly, at a high exposure to available contaminants, nutrient recycling by soil (micro)-organisms is impacted. On-site studies have shown the impact of many pollutant types on micro-organism biomass and diversity, and related enzymatic activities^14–16^. Secondly, as stated above, plant organs responsible for resource capture are less efficient. In addition to roots, when aboveground plant parts are also exposed to contaminant excess, leaf structure can be impacted^17^ and photo-chemical energy production may decrease^18,19^. Thirdly, root association with fungi^20^ and bacteria (see, for instance, ref. ^21^) can be impacted and these mutualistic interactions are pivotal in plant nutrition and water acquisition. Accordingly, mutualistic associations involving adapted fungi and bacteria populations are key players to cope with soil pollution^22,23^. Finally, adaptation to pollution implies trade-offs between the investment of resources to stress tolerance mechanisms and rapid and efficient growth. A cost of adaptation to excessive metal(loid) exposure is commonly reported for plants^24^ and confirmed in a meta-analysis^25^.

To summarise, when contaminant bioavailability increases, available resources for plant growth become more limited. In line with the OPT, our overarching hypothesis is that biomass partitioning should vary plastically along pollution gradients with increasing root exposure to contaminants. Out of the four causes stated above which induce a decrease in available resources, we focused on the disruption of resource uptake by a model leguminous species sensitive to soil pollution (i.e. the dwarf bean, *Phaseolus vulgaris* L., see ref. ^26^) and also on the Symbiotic Nitrogen Fixation (SNF) by root-*Rhizobium* association. As roots are directly exposed to soil pollution, we hypothesised that access to belowground resources is particularly limited, and that root biomass allocation should increase with soil pollution.

To test this hypothesis, we considered two additional important principles. Firstly, the amplitude of plasticity in biomass partitioning varies between species and/or populations. Species having a resource foraging strategy often have a higher level of plasticity (e.g. ref. ^27^). In addition to its sensitivity to soil pollution, this study focuses on a model plant species known for its marked plasticity of biomass partitioning along soil resource gradients^28,29^. Secondly, the biomasses of different plant parts scale allometrically with respect to each other, due to physical and developmental constraints (see ref. ^30^ for the allometry of reproductive organs^13^). Considering belowground and aboveground organs, an allometric relationship can be defined as follows:

1 $${M}_{R}={{\upbeta }}.{M}_{S}^{{{\upalpha }}}$$

where M_R_ represents root biomass, M_s_ is shoot biomass, β is the constant of proportionality, and α is the allometric exponent. Studies of allometric relationships often use the linearised form of Eq. (1):

2 $${{{{{\rm{log }}}}}}\;{M}_{R}={{{{{\rm{log }}}}}}\;{{\upbeta }}+{{\upalpha }}.{{\log }}\;{M}_{S}$$

When α is equal to one the relation is isometric. Otherwise, biomass partitioning between roots and shoots varies allometrically with plant size. In that case, two individual plants in two different environments would differ in organ mass fractions because they have different growth rates and reach different sizes at measurement time. The main caveat in studies of biomass partitioning along environmental gradients is the confusion of simple allometric effects (an allometric relationship exists with α ≠ 1, and change of organ mass fraction is due to the modification of growth rate) with plastic response^31^ consistent with the OPT and requiring a change in the allometric relationship (i.e. a change in the allometric coefficients α or β). In fact, both allometric and plastic responses are often combined^12,32^.

Because toxic effects induced by soil pollution will impact plant growth rate and plant size, it is crucial to delineate allometric and plastic biomass partitioning when studying plant response to pollution stress. To clarify our overarching hypothesis, we suggest three pathways of changes in biomass partitioning that need to be distinguished: (i) a simple allometric change due to a growth rate decrease (toxic effect) combined with an allometric exponent (α) different from 1; (ii) an early delay in root development^9^ (toxic effect), leading to a deficit of belowground parts compared to shoots in the first stages of plant development; and (iii) a plastic increase of biomass partitioning (plant response) in the later development stages in favour of roots to compensate for the lower uptake of soil resources.

In order to have a complete evaluation of these different tenets of biomass partitioning with soil pollution, we aimed at: (i) having an empirical test of our hypothesis and of the different drivers of biomass partitioning; and (ii) performing an in-depth literature survey to verify the ability of former studies to distinguish between the various drivers under investigation. For the empirical study, we created two soil series with increasing Cu and PAH contamination by diluting two contaminated soils with an uncontaminated control soil harvested nearby, with a similar soil texture. These two contaminated soils were collected in a wood preservation site (6 ha, which has been used for over a century to preserve and store timber, posts, and utility poles. The use of creosote, and various Cu-based salts has resulted in soil Cu-contamination over the whole site, and large patches of PAH in smaller areas. The control soil was collected from the grassland next to the site. The addition rates were 0, 1/3, 3/3, and 3/3 of contaminated soil to the control soil for each soil series. Hereafter, these soil series will be referred to as Cu-PAH and HIGH-Cu-PAH relative to their respective contamination levels (Supplementary Table 1). Dwarf bean plants were cultivated on these soils in a glasshouse and harvested at five development stages (from the end of cotyledon opening-stage 1–6 trifoliate leaves-stage 5) to ensure a wide range of plant size in the dataset. To tease apart allometric and plastic biomass partitioning, we fit the different allometric relationships in the different soil treatments with standard major axis regression and we scrutinised potential changes in allometric coefficients (α or β) between root and shoot biomasses (Eqs. 1 and 2, see methods for more details). Similarly, analyses of allometric relationships between root and shoot areas offered a complementary functional view of the equilibrium between resource capture surfaces. Several indicators of plant ability to capture resources (i.e. the amount of water transpired, leaf chlorophyll and nitrogen concentration, and a number of root nodules) were also measured.

Regarding our second objective, we gathered published studies focusing on changes in biomass partitioning when plants are exposed to diverse contaminants in soils. This subject has been investigated in former studies because the development of root and shoot parts is of direct interest regarding the phytomanagement of polluted sites (e.g. ref. ^33^). The main indicator of biomass partitioning available in these studies is the M_R_: M_S_ ratio (see, for instance, ref. ^34^). We performed a meta-analysis of the changes for this ratio according to several factors (e.g. contaminant type, plant functional type—grasses, forbs, and woody plants) to detect any constant pattern in plant response to soil pollution. We checked the dependence of M_R_: M_S_ changes observed with soil pollution and changes in plant size because this dependence would suggest that allometric effects could be involved. Additionally, we modelled the changes in root: shoot ratio according to the different drivers of biomass partitioning characterised empirically (first part of the study). This enabled us to understand the underlying mechanisms producing different M_R_: M_S_, and to decide whether former studies were able to separate the direct impact of soil pollution (toxic effects) from plant response to the changes in resource availability. While the empirical study was performed with Cu and PAH as contaminants, the meta-analysis and the modelling approach enabled enlarging our view regarding biomass partitioning on polluted soils. Finally, we envisaged future directions of research to improve our understanding of plant response to soil pollution.

## Results

### Plant growth rate

For given soil treatment, homogeneous plant growth rates at all development stages indicate homogeneous conditions for growth during the experiment, apart from soil toxicity (Table 1). The plant biomass at a given development stage and the time to reach this stage decreased according to the proportion of contaminated soil, and with a higher decrease on the HIGH-Cu-PAH gradient (Table 1). As a result, soil phytotoxicity based on the decrease in plant growth rates can be ordered as follows: 1/3 Cu-PAH (−16% considering all development stages) <2/3 Cu-PAH (−37%) <Cu-PAH (−53%) <1/3 HIGH-Cu-PAH (−65%) <2/3 HIGH-Cu-PAH (−83%) <HIGH-Cu-PAH (−91%). Note that the response on 1/3 Cu-PAH soil was often not statistically different from the control soil. On the HIGH-Cu-PAH soils, most plants (21 out of 24 plants) did not develop and were harvested at the first cotyledon stage.

**Table 1:** Growth performance of *Phaseolus vulgaris* plants across the soil series, and at different development stages.

Stage	Soil	N	Time to grow (days)	Plant biomass (g)	Growth rate (g.day^−1^)
1	Control	5	12.6 ± 0.4 ab	0.335 ± 0.048 c	0.027 ± 0.004 c
1	1/3 Cu-PAH	5	10.6 ± 2.6 a	0.308 ± 0.013 bc	0.034 ± 0.005 c
1	2/3 Cu-PAH	5	20.3 ± 6.7 bc	0.269 ± 0.041 bc	0.020 ± 0.012 bc
1	Cu-PAH	5	20.6 ± 0.4 c	0.278 ± 0.024 bc	0.014 ± 0.001 b
1	1/3 HIGH-Cu-PAH	5	34.4 ± 2.1 d	0.286 ± 0.026 bc	0.008 ± 0.001 ab
1	2/3 HIGH-Cu-PAH	4	34.8 ± 3.0 d	0.200 ± 0.015 ab	0.006 ± 0,000 ab
1	HIGH-Cu-PAH	21	64.7 ± 3.0 e	0.153 ± 0.007 ab	0.003 ± 0.000 a
2	Control	4	23.5 ± 1.8 a	0.770 ± 0.044 cd	0.033 ± 0.002 d
2	1/3 Cu-PAH	5	36.8 ± 1.9 b	0.690 ± 0.116 cd	0.019 ± 0.005 c
2	2/3 Cu-PAH	4	36.3 ± 1.9 b	0.829 ± 0.066 d	0.023 ± 0.002 cd
2	Cu-PAH	5	40.2 ± 2.1 b	0.607 ± 0.068 bcd	0.015 ± 0.002 bc
2	1/3 HIGH-Cu-PAH	5	41.2 ± 1.2 b	0.496 ± 0.038 ac	0.012 ± 0.001 ac
2	2/3 HIGH-Cu-PAH	4	66.0 ± 3.5 c	0.321 ± 0.043 ab	0.005 ± 0.001 ab
2	HIGH-Cu-PAH	3	71.7 ± 2.7 c	0.237 ± 0.022 a	0.003 ± 0.000 a
3	Control	7	50.1 ± 2.9 a	1.736 ± 0.136 d	0.035 ± 0.002 e
3	1/3 Cu-PAH	6	50.2 ± 2.9 a	1.286 ± 0.084 c	0.026 ± 0.003 d
3	2/3 Cu-PAH	6	55.2 ± 6.7 a	1.117 ± 0.067 bc	0.021 ± 0.002 cd
3	Cu-PAH	6	58.0 ± 6.1 ab	1.021 ± 0.065 bc	0.018 ± 0.001 bc
3	1/3 HIGH-Cu-PAH	10	68.3 ± 4.3 bc	0.869 ± 0.084 b	0.013 ± 0.001 b
3	2/3 HIGH-Cu-PAH	17	76.0 ± 3.3 c	0.469 ± 0.024 a	0.006 ± 0.000 a
3	HIGH-Cu-PAH	1	77.0	0.243	0.003
4	Control	4	72.5 ± 2.1	2.121 ± 0.263 b	0.030 ± 0.004 b
4	1/3 Cu-PAH	5	74.3 ± 5.3	1.894 ± 0.115 b	0.026 ± 0.003 b
4	2/3 Cu-PAH	10	78.1 ± 2.7	1.303 ± 0.100 a	0.017 ± 0.001 a
4	Cu-PAH	8	86.5 ± 1.0	1.137 ± 0.098 a	0.013 ± 0.001 a
4	1/3 HIGH-Cu-PAH	5	76.0 ± 3.8	0.991 ± 0.110 a	0.011 ± 0.002 a
4	2/3 HIGH-Cu-PAH	0			
4	HIGH-Cu-PAH	0			
5	Control	5	76.2 ± 1.4	2.742 ± 0.131	0.036 ± 0.002
5	1/3 Cu-PAH	4	83.0 ± 3.8	2.498 ± 0.209	0.030 ± 0.005
5	2/3 Cu-PAH	0			
5	Cu-PAH	1	82.0	1.207	0.015
5	1/3 HIGH-Cu-PAH	0			
5	2/3 HIGH-Cu-PAH	0			
5	HIGH-Cu-PAH	0			

Mean and standard errors are shown and the number of individuals harvested (*N*) indicated. Note that in toxic soils, most plants could not reach the last development stages as planned. They were classified according to their real development stages when harvested by the end of the experiment (e.g. 21 of 25 plants for the High-Cu-PAH soil only reached the first stage). When the influence of soil treatment on measured variables is significant (ANOVA), significant differences between treatments are shown by different letters (post-hoc Tukey pairwise comparisons).

### Specific leaf and root areas

**Fig. 1:**
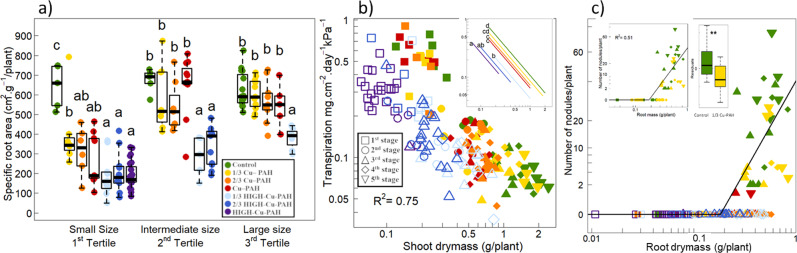
Effect of soil treatments on root development and the capture of belowground resources. In all panels, soil treatments are indicated by different symbol colours. In panel **b**, **c**, development stages are indicated by different symbols (see legend in panel **b**). **a** Impact on Specific Root Area (SRA). Because the response was influenced by plant size, all plants were split into three groups according to root biomasses tertiles. Because of the toxic effect on plant growth (Table 1), plants from the highest phytotoxic soils (2/3 HIGH-Cu-PAH and HIGH-Cu-PAH) are not represented in the highest size class (third tertile), and plants from the HIGH-Cu-PAH soil are not represented in the intermediate size class (second tertile). For each size group, ANOVA were performed to detect differences of SRA with soil treatments. Different letters indicate significant difference between soil treatments (post-hoc Tukey pairwise comparisons). **b** Size-dependent transpiration influenced by soil treatment. Results of the ANCOVA show a strong influence of soil treatments on the intercept of the relationship between shoot biomass and transpiration (*P* < 0.001). Modelled relationships are shown in the top right inset where different letters indicate significant differences of intercept (post-hoc Tukey pairwise comparisons). **c** Size-dependent relationship between root biomass and the total number of nodules. The segmented regression shown is highly significant (*P* < 0.001 for the existence of a breakpoint, Davies test) indicating an absence of nodulation when root mass is inferior to 0.19 g, then a positive relationship with an increasing number of nodules with root mass. Note that nodule number was square root-transformed. In the top left inset, the same segmented relationship is shown for plants growing in the control and the 1/3 Cu-PAH soils. Residuals of the increasing relationship (on the right of the breakpoint) are shown (boxplot in the top left inset), with higher residuals for the control soil (*P* < 0.01, two sample *t*-test). See Supplementary Table 3 for corresponding degrees of freedom and associated *p*-values.

Specific Leaf Area (SLA, cm^2^.g^−1^) varied slightly in our experiment, but differences were not consistent with the dilution rate of contaminated soils (Supplementary Fig. 1a). Additionally, plants with small root biomass showed a clear decrease in Specific Root Area (SRA, cm^2^.g^−1^). This decrease was ordered consistently with the proportion of contaminated soil in the treatments and was higher for the HIGH-Cu-PAH soil series (Fig. 1a, average SRA decrease of 69% compared with the control). For plants of intermediate and high root biomass, plants growing on the Cu-PAH series displayed SRA similar to the control, while plants growing on the HIGH-Cu-PAH series still showed lower SRA (Fig. 1a).

### Resource capture

The amount of water taken up and transpired decreased strongly with bean shoot size (Fig. 1b). This decrease was observed for all plants, even for small ones harvested at the first development stages. In addition, we found a clear decrease due to soil treatments, as shown by the decrease in the intercept of the size-dependent relationship (Fig. 1b). The decrease was ordered consistently with the ecotoxic impact observed on plant growth: plants in the HIGH-Cu-PAH treatment were significantly more affected than those in the Cu-PAH treatment. The lowest intercept for the HIGH-Cu-PAH soil corresponded to a maximum decrease of 63% of water transpiration compared to the control. This decrease on the 1/3 Cu-PAH soil was not statistically different.

As to Nitrogen uptake, apart from a strong size-dependent relationship between leaf N concentration and plant size, we did not observe any additional effect of soil treatments (Supplementary Fig. 1b). Root nodulation did not start below a root mass threshold of 0.19 g (Fig. 1c). Thus, roots from the HIGH-Cu-PAH soil series did not display nodules. Root nodulation mostly occurred for plants growing in the control soil and the 1/3 Cu-PAH soil, from development stages 3–5, with a significantly higher number of nodules for the control soil (Fig. 1c).

Finally, regarding light capture and chlorophyll synthesis, the different soil treatments did not impact chlorophyll content consistently with soil contamination. More details are provided in Supplementary Fig. 1c, d.

### Allometric relationships

**Fig. 2:**
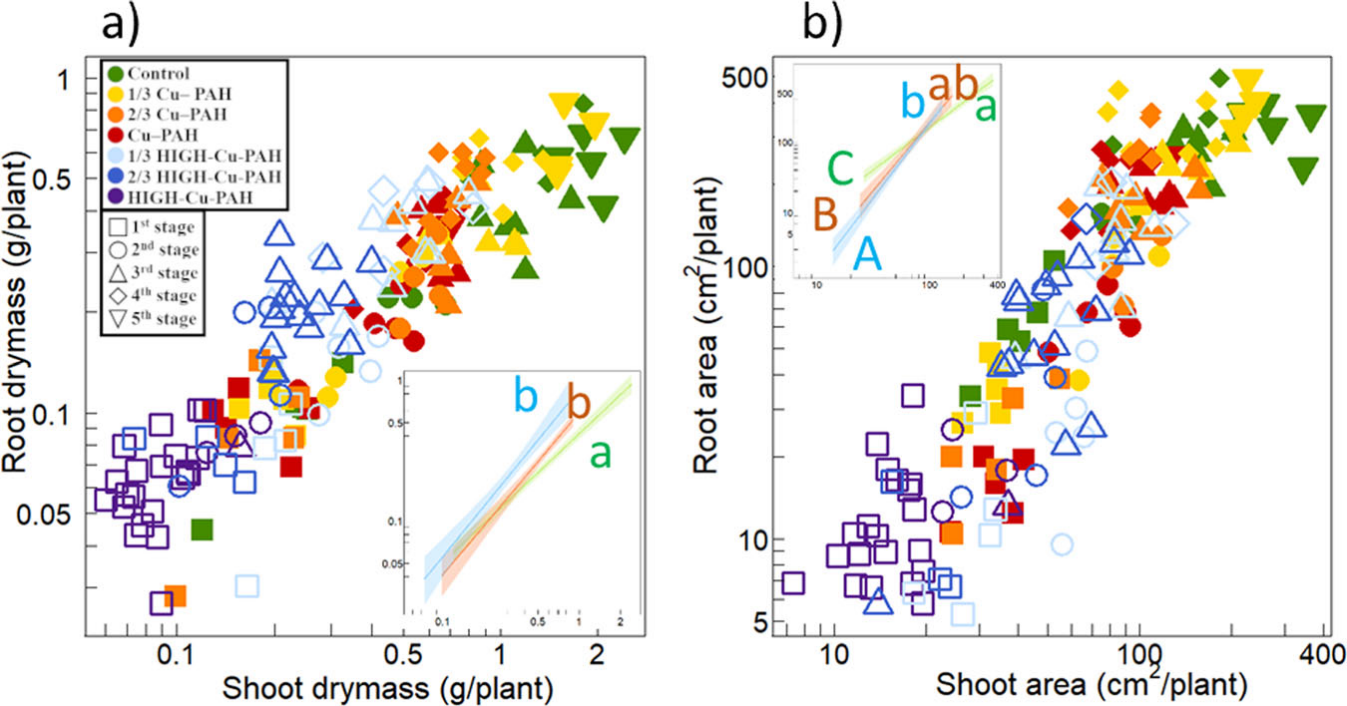
Influence of soil treatment on the allometric relationships between root and shoot biomass and area. Symbols and colors are as in Fig. 1 (see legend in panel **a**). Here, for the sake of clarity, relationships for the different soil treatments are presented in three groups in the insets of both panels (Control and 1/3 Cu-PAH soils in green; 2/3 Cu-PAH and Cu-PAH soils in red; 1/3 HIGH-Cu-PAH and 2/3 HIGH-Cu-PAH soils in blue). Corresponding relationships are similar for both soil treatments of the same group (Supplementary Table 2). Fitted relationships (lines) and 95% confidence envelopes (shaded areas) are shown except for plants from the HIGH-Cu-PAH soil, because they did not grow. Lowercase letters indicate difference in α allometric coefficient (*P* < 0.05). Uppercase letters indicate difference in β proportionality coefficient.

Allometric relationships could not be observed for plants on HIGH-Cu-PAH soils because they did not grow during our experiment (Table 1). Compared to the control, plants on 1/3 Cu-PAH soil did not differ in their allometric relationship (Fig. 2, Supplementary Table 2). For other soil treatments, modifications of allometric relationships are explained below.

Changes in β (constant of proportionality): Relationships between root and shoot biomasses did not show changes in β (Supplementary Table 2a, Fig. 2a), indicating that small plants on polluted soils did not show a deficit of roots compared to the control (see also Supplementary Fig. 2a showing the variation of root mass fraction with plant size). Conversely, a change of β was seen for root and shoot areas (Supplementary Table 2b, Fig. 2b), with small plants having a strong deficit of root surface (see also Supplementary Fig. 2b showing the variation of root area fraction with plant size).

Changes in α (allometric exponent). The α of dwarf beans was inferior to 1 (0.92) in the control soil. Plants in contaminated soils showed an increase of α above 1, both in terms of biomass (Fig. 2a, Supplementary Table 2a) and area (Fig. 2b, Supplementary Table 2b). α increased with contamination until the 1/3 HIGH-Cu-PAH treatment (Supplementary Table 2). This increase in α is more visible when considering the relationship for areas (Supplementary Table 2b, Fig. 2b) rather than for biomass (Supplementary Table 2b, Fig. 2b).

### Meta-analysis of M_R_: M_S_ with soil pollution

**Fig. 3:**
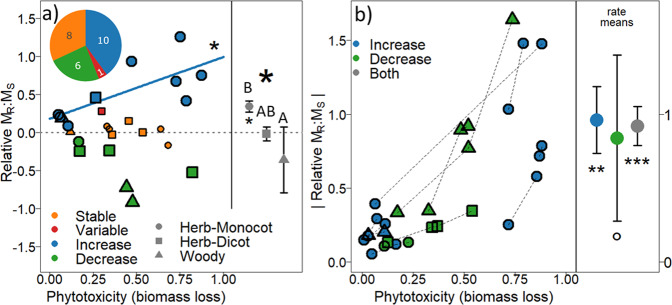
Results of the meta-analysis of published results regarding biomass partitioning on polluted soils. **a** Effect size on M_R_:M_S_ in regards of phytotoxicity (normalised biomass loss) for the different case studies. For each case study, the response on all polluted soils were averaged before calculating a single value for the relative M_R_:M_S_ and phytotoxicity. The classification of the M_R_:M_S_ response is shown: stable (orange); variable (red); increase (blue); and decrease (green). The number of case studies found for each class is shown in the top left pie chart. Different plant functional groups are distinguished with different symbols. The amplitude of changes in M_R_:M_S_ is related to the amplitude of phytotoxicity for case studies corresponding to the “increase” class (*P < 0.05). On the right-hand side of the panel, mean (±se) of relative M_R_:M_S_ are shown for the three plant functional groups. The functional group has a significant effect on relative M_R_:M_S_ (ANOVA, *P < 0.05). Different letters indicate a significant difference between groups (*P* < 0.05 after post-hoc pairwise multiple comparisons). Wilcoxon tests were performed for each group to test difference from zero (*P < 0.05). See Supplementary Table 3 for corresponding degrees of freedom and associated *p*-values. **b** Effect size on M_R_:M_S_ regarding phytotoxicity within each case study having at least two different polluted soils and showing an increase or decrease of M_R_:M_s_. Note that relative M_R_:M_S_ are shown in absolute values. Responses from the same case study are linked with dotted lines. Symbols (circle, square, and triangle) are as in (**a**). The rate of change (Δ M_R_: M_S_ / Δ biomass loss) is calculated for each case study. Mean rates and their confidence intervals (CI_95%_) are shown. Wilcoxon tests (***P < 0.001; **P < 0.01; °*P* < 0.1) were used to test the difference of rate means from zero.

The main contaminants involved in the case studies considered were metal(loid)s (15 case studies) and organic contaminants such as petroleum and derivatives (10 case studies). These case studies concerned 13 monocotyledon species, 8 forbs (dicotyledonous herbs) and 4 woody species (Table 2). Ten cases reported an increase of *M*_*R*_*: M*_*S*_; six cases a decrease, eight cases a stable response, and one case a variable response (Fig. 3a). For the 10 cases of *M*_*R*_*: M*_*S*_ increase, the strength of the response was related to that of biomass reduction (Fig. 3a). Changes in *M*_*R*_*: M*_*S*_ were influenced by the type of plant species involved. Monocotyledonous species showed a significant increase in their *M*_*R*_*: M*_*S*_; (relative response > 0), while woody tree species showed negative values (though not significantly different from 0). Forbs had an intermediate response (Fig. 3a). We did not find any significant effect on relative *M*_*R*_*: M*_*S*_ for all the other factors recorded. For case studies including at least two treatments with polluted soils, the strength of the *M*_*R*_*: M*_*S*_ response was related to the decrease in plant growth due to contamination (Fig. 3b). Noticeably, in 22 out of the 25 case studies, the method to estimate the biomass of plant parts and *M*_*R*_*: M*_*S*_ was a single harvest after a similar growth period for all soil treatments. For the three remaining case studies (from the same study), two harvest times were realised, without accounting for plant size.

**Table 2:** Description of the case studies considered in the meta-analysis of root: shoot ratio response to soil pollution.

Case study	Species	Contaminant	Family	Plant type	Life cycle	Root: shoot response	Harvest time	Rationale	Focus on biomass allocation	Climate (Köppen classification)	Country	Reference
1	*Sasaella glabra*	Lead	Poaceae (Bambusoideae)	Herb-Monocot	Perennial	Increase	One harvest at 9 months	Biomass allocation and lead accumulation in different organs	Yes	Dry-winter Humid subtropical (Cwa)	China	^55^
2	*Sasa fortunei*	Lead	Poaceae (Bambusoideae)	Herb-Monocot	Perennial	Decrease						
3	*Sasa auricoma*	Lead	Poaceae (Bambusoideae)	Herb-Monocot	Perennial	Increase						
4	*Shibataea lanceifolia*	Lead	Poaceae (Bambusoideae)	Herb-Monocot	Perennial	Increase						
5	*Rumex dentatus*	Copper	Polygonaceae	Herb-Dicot	Annual	Stable	One harvest at 2 months	Growth and copper accumulation between metallicolous and non-metallicolous populations	No	Humid subtropical (Cfa)	China	Liu et al. 2004
6	*Brachiaria brizantha*	Petroleum	Poaceae	Herb-Monocot	Perennial	Stable	Two harvests (90 and 180 days)	Species development for phytoremediation	No	Tropical savana (Aw)	Venezuela	Merkl et al. 2005
7	*Cyperus aggregatus*	Petroleum	Cyperaceae	Herb-Monocot	Perennial	Increase						
8	*Eleusine indica*	Petroleum	Poaceae	Herb-Monocot	Annual	Stable						
9	*Phragmites australis*	Petroleum	Poaceae	Herb-Monocot	Perennial	Increase	One harvest at 2 months	Biomass allocation	Yes	Dry-winter Humid subtropical (Cwa)	China	Nie et al. 2010
10	*Amaranthus paniculatus*	Nickel	Chenopodiaceae	Herb-Dicot	Annual	Decrease	One harvest at 7 days	Biomass allocation and nickel accumulation in different organs	Yes	Mediterranean—Hot summer (Csa)	Italia	Iori et al. 2013
11	*Brassica pekinensis*	Copper	Brassicaceae	Herb-Dicot	Annual	Stable	One harvest at 14 days	Toxicity on plant metabolism, consequence for growth (including Root: Shoot ratio)	Yes	Humid subtropical (Cfa)	China	Xiong et al. 2006
12	*Kummerowia stipulacea*. Metalicolous population	Copper	Fabaceae	Herb-Dicot	Annual	Increase	One harvest at 7 days	Comparison of biomass allocation between metallicolous and non-metallicolous population	Yes	Humid subtropical (Cfa)	China	Zhang et al. 2014
13	*Kummerowia stipulacea*. Unmetalicolous population	Copper	Fabaceae	Herb-Dicot	Annual	Decrease	One harvest at 7 days	Comparison of biomass allocation between metallicolous and non-metallicolous population	Yes	Humid subtropical (Cfa)	China	Zhang et al. 2014
14	*Lythrum salicaria*	Copper	Lythraceae	Herb-Dicot	Perennial	Stable	One harvest at 5 weeks	Plant growth response	Yes	Humid subtropical (Cfa)	United States	Uveges et al. 2002
15	*Zea mays*	Mix metal(loïd)s	Poaceae	Herb-Monocot	Annual	Increase	One harvest at 21 days	Plant growth response	No	Cold semi -Arid (BSk)	Spain	Brennan et al. 2014
16	*Chrysopogon zizaniodes*	Lead	Poaceae	Herb-Monocot	Perennial	Increase	One harvest at 7 months	Species development for phytoremediation and accumulation of contaminant in different organs	No	Humid subtropical (Cfa)	Australia	Danh et al. 2011
17	*Chrysopogon zizaniodes*	Copper	Poaceae	Herb-Monocot	Perennial	Stable						
18	*Ailanthus altissima*	Petroleum	Simaroubaceae	Woody tree	Perennial	Decrease	One harvest at 240 days	Species development for phytoremediation	Yes	Mediterranean—Hot summer (Csa)	Iran	^33^
19	*Fraxinus rotundifolia*	Petroleum	Oleaceae	Woody tree	Perennial	Increase						
20	*Melia azedarach*	Petroleum	Meliaceae	Woody tree	Perennial	Stable						
21	*Triticum aestivum*	Petroleum-coke	Poaceae	Herb-Monocot	Annual	Increase	One harvest at 2 months	Species development for phytoremediation	No	Warm summer continental (Dfb)	Canada	Nakata et al. 2011
22	*Deschampsia flexuosa*	Petroleum-coke	Poaceae	Herb-Monocot	Perennial	Increase						
23	*Leucanthemim vulgare*	Petroleum	Asteraceae	Herb-Dicot	Perennial	Variable	One harvest at 16 weeks	Species development for phytoremediation	Yes	Mediterranean—Hot summer (Csa)	Iran	Noori et al. 2014
24	*Leucanthemim vulgare*	Mix metal(loïd)s	Asteraceae	Herb-Dicot	Perennial	Decrease	One harvest at 2 months	Phytotoxicity of slag containing heavy metals and biomass allocation	Yes	Warm summer continental (Dfb)	Canada	Ryser and Emerson et al. 2007
25	*Salix smithiana*	Mix metal(loïd)s	Salicaceae	Woody tree	Perennial	Decrease	One harvest at 4 years	Biomass allocation and lead accumulation in different organs	Yes	Oceanic (Cfb)	Czech Republic	Vondráčková et al. 2017

Corresponding references are numbered in the last column, and full details of these references are provided in the Supplementary References.

### Modelling of changes in root: shoot ratio due to soil pollution

**Fig. 4:**
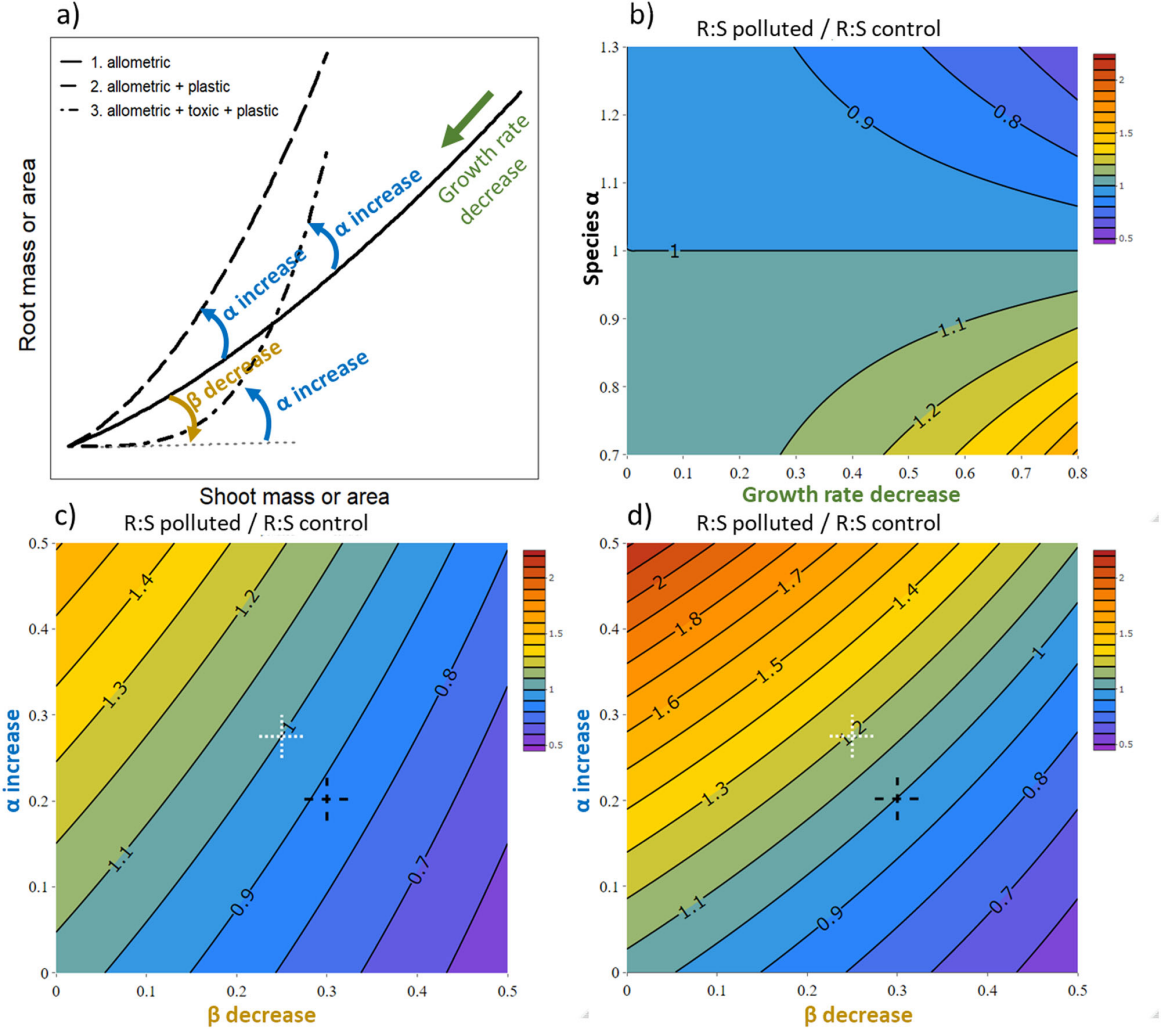
Synthesis of plant response to soil pollution stress and modelling of corresponding changes in root: shoot (R:S) ratio. **a** Different possible plant development trajectories (in natural scale). In all cases, the phytotoxic effect decreases growth rate leading to plants with smaller roots and shoots (green arrow in the top right corner). The baseline development shown has an α coefficient >1. Case 1 (solid line): no plasticity of biomass in response to pollution stress. See panel **b** and Supplementary Fig. 3a-d for consequence on R:S. Case 2 (dashed line): a plastic increase of biomass partitioning (α increase) is involved in response to pollution stress. R:S will be higher for plants exposed to pollution (Fig. 2a for shoot and root biomasses in this study, see also Supplementary Fig. 3e-h). Case 3 (dotted + dashed line): a decrease of early root development is observed (β decrease) for plants exposed to soil pollution, and then a plastic increase of biomass allocation (α increase) occurs to offset the decrease in soil resource acquisition (Fig. 2b in this study for root and shoot areas). See panel **c**, **d** (and Supplementary Fig. 3i-k) for consequences on R:S. **b**–**d** Modelled changes in R:S ratio compared to a control situation (growth in unpolluted soil). Increase and decrease rates are indicated on the different axes (see Eqs. 5 and 6 in the main text). Parameters used for the simulations were as follows: daily shoot growth rate for the control situation: 5 unit.day^−1^; α for control situation: 0.92 (this study); β for control situation: 0.47 (this study); number of day of growth: 10 in (**c**) and 20 in (**d**); growth rate decrease for **c**, **d**: 0.5. Note that different parameter values will produce the same patterns but after different growing durations. **b** Changes in R:S in the absence of plasticity (Case 1 in panel **a**) and according to the species α allometric exponent. **c**, **d** Changes in R:S in case of early negative impact on root development (β decrease), and plastic increase of root allocation (α increase, Case 3, panel **a**) after a short (**c**) and long (**d**) period of growth. Black and white crosses position the same changes of β and α.

We considered three scenarios of increasing complexity to scrutinise potential changes in root: shoot ratio (Fig. 4a, Supplementary Fig. 3a, e, i). The simplest scenario implied a decrease in plant growth rate (toxic effect). If the studied species had an intrinsic allometric coefficient α different from 1, changes in growth rate with pollution would induce changes in the root: shoot ratio in all cases (Fig. 4a, b; Supplementary Fig. 3a-d). The changing strength was imposed by the initial α value and the strength of the growth rate decreased (Fig. 4b), and was independent of the growth period (Supplementary Fig. 3c). When α was inferior to 1 (dwarf bean in this study), the expected result was an increase in root: shoot ratio (Fig. 4b, Supplementary Fig. 3c). This first scenario led to results similar to those found in the literature reporting increase or decrease of root: shoot ratio (Fig. 3a, b): change strength was related to the impact on plant growth (Fig. 4b, Supplementary Fig. 3d).

The second scenario required a growth rate decrease (toxic effect) and a plastic increase of α (plant response), consistent with the response of dwarf bean in this study which considered the biomasses of plant parts. It led to an increase in root: shoot ratio in all cases (Fig. 4a, Supplementary Fig. 3e-h). The third scenario involved a growth rate decrease (toxic effect), a plastic increase of α (plant response) and a decrease of β (toxic effect on initial root development). The net effect on the root: shoot ratio was complex and depended on the relative importance of changes in α and β, and on the duration of the growth period (Fig. 4c, d; Supplementary Fig. 3i–k). An important β decrease could be compensated for by an important α increase, or by a moderate α increase and a longer period of growth. Noticeably, with the same changes in growth rate, α and β, the change in root: shoot ratio could vary with elapsed time, being decreased, unchanged, or increased compared to the control (Fig. 4c, d, Supplementary Fig. 3k).

## Discussion

This study aimed to improve our knowledge of plant growth regarding soil pollution. It was first designed to delineate the toxic effects of soil pollution on biomass partitioning from plant response due to the indirect reduction in resource availability. We found that not only biomass partitioning but also most plant responses depended on plant size. Accordingly, we discuss below the phytotoxic impacts of soil pollution, before concluding with our hypothesis regarding the plasticity of biomass partitioning, and the various drivers involved. The meta-analysis provided complementary results. Several case studies reported changes in biomass partitioning (using the M_R_:M_S_ ratio as a proxy) and depended on plant functional type. However, modelling the root: shoot ratio (consistently with the empirical part of this study) confirmed that considering both the toxic effects of soil pollution and plant response is essential to the interpretation of changes in biomass partitioning. If studies continue to be designed without considering this distinction, our knowledge regarding the determinism of this response will remain blurred. Based on this empirical, literature-reviewed, and modelling work, we were finally able to define future directions to help our understanding of plant growth in response to soil pollution.

Regarding phytotoxic impacts of soil pollution, the dramatic decrease of plant growth found here, organised according to the soil dilution rate and the level of soil contamination confirmed the increasing phytotoxicity along our experimental gradients. The toxicity of the HIGH-Cu-PAH soil was so high that plant growth was almost impossible beyond the opening of the cotyledons. Indeed, reduction of plant growth for some sensitive plant species, including dwarf beans, is used as a bioassay to reveal soil phytotoxicity^26^. Additionally, changes in the SLA and SRA values can indicate a plastic response of plants along resource gradients, but in our case, SLA was not modified. In response to a potential decrease of available soil resources with pollution, an allocative response would imply an increase in SRA. Instead, the large decrease of SRA highlighted a soil phytotoxic effect, consistent with a decrease in root elongation and of the proportion of fine roots reported in the case of exposure to diverse soil contaminants, including PAH^6^ and Cu^8^. In a second phase, secondary lateral root growth is often observed in response to contaminant exposure^35,36^. This could explain the recovery of SRA for intermediate to large-sized plants growing in the Cu-PAH soil series. For plants in the HIGH-Cu-PAH soil series, root growth was too limited for such a recovery response to occur at all.

As to changes in resource capture and uptake, leaf N concentration of legumes depends on both N availability in soil and symbiotic fixation^37^. Here, the greatest source of leaf N variation was a dilution effect with plant size (Supplementary Fig. 1b). Additionally, the main impacts of soil pollution on SNF were: (i) a decrease in plant growth, with most plants that did not reach a root size threshold necessary to start SNF; (ii) for the biggest plants on the control and 1/3 Cu-PAH soils, SNF occurred and soil pollution decreased root nodulation as shown in the previous studies^21^. Furthermore, chlorophyll synthesis is a pre-requisite to produce photo-chemical energy. The relationship between chlorophyll concentration and plant size was similar to that of leaf N concentration with plant size. Our results did not suggest a quantitative impact of the pollution gradients on photosystem synthesis (more details provided in Supplementary Fig. 1 and related comments). Regarding water uptake and transpiration, we found a distinct decrease in transpiration per unit of leaf area with plant shoot size. This decrease was observed even for the smallest plants (0.03–0.5 g of roots) in pots containing 800 g of soil, so inter-root competition cannot explain this result. Instead, we suggest that lower leaf N and chlorophyll concentrations observed with plant growth correspond to lower concentrations of Ribulose-1,5-bisphosphate carboxylase/oxygenase (RuBCPase, a N-rich protein), a lower photosynthetic capacity and C assimilation, and thus transpiration^38,39^. Lower leaf N concentrations of legumes also involve lower stomatal conductance^40^. Alternatively, root hydraulic conductivity may decrease with bean root age^41,42^. Whatever the reason for this decrease in plant size, we found an additional decrease in transpiration along our pollution gradients (Fig. 1b). An impact on root morphology and development is one of the processes explaining the reduction of water uptake and transpiration for plants exposed to metal stress^9^. This loss of root function is also reported for plants exposed to PAH, those plants being described as water stressed^6^. It offers a simple interpretation of our results. A complementary interpretation is related to the deficit of root area compared with shoot area for small plants on polluted soils (Fig. 2b; Supplementary Fig. 2b): despite a similar water sink (leaf and shoot areas), the water source (root area) is depleted.

In conclusion regarding the acquisition of resources, after considering plant-size effects, we found a clear decrease in water transpiration with soil pollution, as well as a decrease in SNF for the biggest plants. These results were consistent with our hypothesis that access to the soil resources would be more limited because roots are directly exposed to soil contaminants. These results shed new light on the similarity observed between root response in cases of water stress and metal stress^43^.

Regarding biomass partitioning, two distinct changes in allometric relationships were observed with soil pollution. The decrease of β (when the surfaces of plant parts are considered) is directly related to the aforementioned toxic effect on roots and SRA. The increase of α (considering both the biomass and area of plant parts) validate our hypothesis: plant response includes an increase in biomass partitioning in favour of roots when plants are exposed to soil pollution. Gathering results about resource capture and allometric relationships, we propose the following explanation as to the most likely to explain our results. In the earliest stages of development, a deficit in root area was due to the strong toxic effect of soil pollution on SRA. This probably explained the transpiration deficit observed for plants growing in polluted soils. Plant response involved two mechanisms: (i) an increase of biomass allocation towards roots as plants continued to grow; and (ii) recovery of SRA during a secondary phase of root growth. Both responses enabled recovery of the equilibrium between root and shoot areas corresponding to that of the control for bigger plants (see also Supplementary Fig. 2b). This plastic response (α increase) was stronger as the soil contamination increased (for the Cu-PAH soil series, and for the 1/3 HIGH-Cu-PAH soils), while it did not increase further for the 2/3 HIGH-Cu-PAH soils (Supplementary Table 2). This may show an upper critical threshold in plant ability to respond to pollution stress. Similarly, plants on the most phytotoxic soil (the HIGH-Cu-PAH soil) did not grow, and no plastic response could take place.

To conclude our first objective, we confirmed that biomass partitioning of the model species during its growth resulted from (1) an allometric scaling exponent α different from (1) combined with a growth rate decrease, (2) the impact on early root development (β decrease), and (3) its ability to recover to limit the loss in resource acquisition (plastic change of α). The expected β decrease is only evidenced here when considering root and shoot surfaces. Measurements made earlier, just after seed germination, may have been more appropriate to detect this effect. Very early root development is indeed a good indicator of soil phytotoxicity and is used in bioassay studies^44^. The distinction between allometric and plastic biomass partitioning is an important question in the ecological literature to understand plant adaptation to different ecological stresses^45^. Overall, our results confirmed that this essential distinction is valid in polluted environments.

The meta-analysis of published results and comparison with the modelling approach provided additional insights regarding biomass partitioning of plants under soil pollution stress. At first glance, the increase of M_R_:M_S_ for monocotyledonous species reported in many case studies would be consistent with a plastic increase of biomass partitioning due to soil pollution. However, we can pinpoint several arguments explaining that simple allometric effects can also be at stake. Several studies have reported that most grass species have an allometric coefficient α lower than 1 (their M_R_:M_S_ decreases as a plant grows)^13,46^, possibly to compensate for a lower nitrogen uptake per root mass^47,48^. In this situation, the higher the decrease of the growth rate, the higher the increase of the M_R_:M_S_ ratio due to allometric effects (see Fig. 4b). This is true when comparing several plant species all having allometric coefficients lower than 1 (as expected for grass species, Fig. 3a, see the similarity with Supplementary Fig. 3d) or at the intra-specific level where increased phytotoxicity led to stronger biomass loss and higher M_R_:M_S_ (Fig. 3b, see the similarity with Supplementary Fig. 2d). Similarly, woody species often show an allometric coefficient α higher than 1^13^. Simple allometric effects can also be involved in results reporting a decrease in M_R_:M_S_ for those species.

Finally, the determinism of biomass partitioning highlighted by our empirical results is complex. The corresponding modelling approach showed that all kinds of results reported in the literature (increase, decrease, stability, and variability of M_R_:M_S_, Fig. 3a) can result from: (i) varying parameters of allometric relationships (α and β), and (ii) constant allometric relationships but after different growth periods (Fig. 4c, d). Note that our modelling approach was based on standard allometric relationships (Eq. 1 and its linear form in Eq. 2). Indeed, linear log–log relationships are the most common in published studies, and our empirical approach showed linear relationships consistent with these standard equations (Fig. 2). Still, because some studies shown exceptions where log–log relationships are not strictly linear^49^, we further investigated the consequences of more complex relationships where the scaling relationship between root and shoot vary with plant size. Results and interpretations remained unchanged and consistent (See Supplementary Fig. 4 and related comments).

Despite the importance of biomass partitioning both in understanding plant response to soil pollution and in designing efficient phytomanagement options, none of the 25 case studies investigated here was able to explain what drove the response of M_R_:M_S_. Reported changes can be purely allometric, and reported stable responses could hide complex effects including plasticity of biomass partitioning. We encourage future studies to broaden their scope beyond the uninformative measurement of plant root and shoot development at a single date after only one period of growth.

The necessary distinction between pollution effect and plant response proposed here led us to raise four future directions of research, all related to biomass partitioning.

First direction: How general is the plastic response to soil pollution observed here? From our results, we suggest that similar plant responses should be detected for plants or genotypes sensitive to soil pollution until an excessive exposure to contaminants breaks any possible plastic response. Regarding plants tolerant to contaminant-induced stress, their access to soil resources should be guaranteed by a more resistant plant root system, potentially associated with mutualist fungi^50^. Consistently with the ecological theory, we expect these stress-tolerant plants to show a lower impact on their growth rate and a lower level of plasticity^51^.

Second direction: Does published literature contain useful information regarding changes in biomass partitioning due to soil pollution? Former studies suffer from methodological issues as stated above. However, considering the large body of literature dedicated to planting response related to soil pollution, other studies not specifically focused on this subject certainly contain useful information, as long as shoot and root parts have been quantified at distinct development stages (see, for instance, ref. ^52^). Such studies could serve as a good base for future meta-analyses.

Third direction: How are other components of plant biomass partitioning (especially reproductive allocation) affected by soil pollution? Reproductive allocation is directly related to plant fitness. Studies of reproductive allocation in response to soil pollution should also consider the complex distinction between simple allometric effect and plastic response of reproductive allocation^30,53^. Otherwise, the same methodological pitfalls will arise (see, for instance, ref. ^54^).

Fourth direction: What is the relative contribution of allometry and plasticity to the accumulation of contaminants in plant parts? Biomass partitioning studies in the context of soil pollution also aim to determine contaminant transfer and accumulation in different plant parts^18,55^. This is especially important for metal(oids) because adapted plants show contrasting strategies regarding metal accumulation^56^. For instance, changes in metal hyper-accumulation by some metallicolous plant species are known to vary with metal availability in soils^57^. If plant growth rate varies simultaneously, this change could be partly due to allometric effects. Indeed, accumulation in plant tissues has been shown to depend on plant size (e.g. ref. ^58^). Delineating allometric and plastic determinism of metal(oid) accumulation should be important both for studies of plant adaptation to metal stress and for phytomanagement scenarios based on phyto-accumulation of contaminants in plant parts.

In conclusion, this study demonstrates the relevance of ecological theory to explain plant responses in polluted systems, providing a more accurate methodology (studies of plant allometry and plasticity) and concepts (the distinction between effect and response, optimal biomass partitioning). By doing this, we confirmed that optimal partitioning is part of the plant response to soil pollution, but also that biomass partitioning is driven by other effects due to soil pollution. These results stress the similarity of functioning between a soil pollution gradient and a soil resource gradient. Given the solid background of ecological theory regarding plant response along resource gradients, this should open new research areas for plant ecologists motivated to investigate the functioning of polluted ecosystems.

## Materials and methods

### Pollution gradients, soil series, and glasshouse conditions for the empirical study

Soils used for this experiment were collected from a wood preservation site (6 ha). In this site, the use of creosote, and various Cu-based salts has resulted in soil Cu-contamination over the whole site and large patches of PAH in smaller areas^59^. Former studies have shown the ecotoxic impact of this contamination on vegetation biomass, cover, richness and diversity^60^ and the reduction of soil enzymatic activity^61^. In February 2016, 65 kg of soil were collected from two areas of the site, one known for its contamination in Cu, and the other previously identified for its contamination in PAH^59^. An additional control soil was collected from the grassland next to the site. The control corresponds to an alluvial sandy soil (Fluviosol - Eutric Gleysols, World Reference Base for soil resources) and the contaminated soils were developed on this Fluviosol. Soils were transferred to a glasshouse nearby and spread out thinly on a tarpaulin for 15 days to ensure complete air drying. Ten samples of each soil were analysed for their PAH, Cu, C, N, and P concentrations. N concentration was higher in the control soils, while polluted soils showed higher P-availability (Supplementary Table 1). Regarding PAH, the 16 regulatory PAH were quantified. The range of soil properties and contamination values (889 ± 10 mg Cu.kg_soil_^−1^ and 657 ± 331 mg PAH.kg_soil_^−1^ for the first contaminated soil, 4276 ± 209 mg Cu.kg^−1^ and 3142 ± 419 mg PAH.kg_soil_^−1^ for the second contaminated soil) showed Cu and PAH contamination in both cases, with higher contamination of the second soil. In this study, these soils are referred to as Cu-PAH soil and HIGH-Cu-PAH soil, respectively.

To create the soil series, both soils were mixed by combining one third and two-thirds of air-dried contaminated soils with the control soil (March 2016) giving seven soil treatments: Control, 1/3 Cu-PAH, 2/3 Cu-PAH, Cu-PAH, 1/3 HIGH-Cu-PAH, 2/3 HIGH-Cu-PAH, and HIGH-Cu-PAH. Each of these seven soils was divided into 25 pots (10 × 10 × 15 cm) containing 800 mg of soil, giving a total of 175 pots. In order to inoculate all potted soils with similar micro-organism populations (especially *Rhizobium* populations), 1 g of control soil was added to the pots with undiluted polluted soils and vice versa. All pots were watered and weighed to determine their water holding capacity and left for 2 weeks to enable micro-organisms populations to react.

To ensure that the environment was as homogeneous as possible during the whole experiment, a whitened glasshouse was used to favour diffuse and homogeneous solar radiation, and to limit differences in temperature and Vapour Pressure Deficit (VPD). In the case of a temperature increase above 25 °C, the glasshouse was also cooled by automatic ventilation and misting was used to avoid an increase of VPD above 1 kPa. An air temperature and humidity probe (U23 Prov V2 ®Hobo) was used to monitor VPD variations (kPa) during the whole course of the experiment.

### Plant cultivation and monitoring of plant development

The dwarf bean (*Phaseolus vulgaris*, cv. Oxinel, *® Vilmorin*) was chosen as a model plant species because of its known plasticity of biomass allocation, both for wild and selected genotypes^29^. This plasticity has been detected in response to soil resources^29^ and also light regimes^62^. In addition, it is a species commonly used as bio-assays in ecotoxicology due to its sensitivity to soil pollution (see ref. ^26^ using soils from the same site as this study). Seeds of similar weight [0.22; 0.30 g] were selected to avoid large differences in seed reserves. After soaking for 4 h in tap water, three seeds were sown in each of the pots on March 21. Germination took 11–16.2 days depending on the soil treatment and this time increased with soil contamination. As a large majority of the seeds germinated, 1 seedling per pot was selected randomly and kept for the experiment. For each soil, we planned to harvest five plants at five different development stages (stage 1: end of cotyledon opening, stage 2: first trifoliate leaf, 3: second trifoliate leaf, 4: 3–4 trifoliate leaves, 5: 5–6 trifoliate leaves) giving 25 plants for each soil. Pots were watered every 2 or 3 days and weighed to maintain the water holding capacity (WHC) of soil at 60%^63^. Plants were harvested for analysis when they reached the desired development stage. In most phytotoxic soils, plants did not reach the fourth or fifth stage by the end of the experiment, thus they were harvested and classified into their real development stage at harvest (see for instance Table 1 shows that most plants of the High-Cu-PAH soil did not grow and were classified in development stage 1).

### Biomass partitioning, root and shoot (specific) areas

On the day of harvest, plant parts were separated (stem, leaves, and roots). Roots were washed gently with water and nodule numbers were counted. All organs were scanned and analysed to determine their area (software Winfolia for leaves and stems, WinRhizo for roots, Regents Instruments, Quebec, Canada). Then all plant samples were dried and weighed. The whole process determined the dry biomass of plant parts, their area, as well as Specific Leaf Area (SLA, cm^2^.g^−1^) and Specific Root Area (SRA, cm^2^.g^−1^). Analysis of SLA and SRA is important because: (i) they may also be involved in plant response along resource gradients to maintain a functional equilibrium. For instance, SLA can increase strength in the shade to maintain light capture area^13^, and SRA can increase to maintain water uptake during water stress^64^; and (ii) they may be impacted by soil pollution. A decrease in SRA is part of the root syndrome in phytotoxic soils because of decreasing root elongation and root thickening^43^.

### Indicators of resource acquisition

To estimate light capture and potential acquisition of photo-chemical energy, we assessed chlorophyll a, b and another carotenoid synthesis by determining their leaf concentrations (See Supplementary Information for more details regarding corresponding methods).

Water uptake and transpiration: To limit water evaporation, the soil in each pot was covered with a small plastic sheet (10 × 10 cm). At each watering, the mass of water added to maintain the pot at 60% of SWHC was recorded as the amount of water taken up and transpired since the last watering. The last 10 days before harvest were considered for analysis of plant transpiration. Independently of soil treatments, the amount of water transpired could be impacted by the leaf area (and the number of stomata), and by the variation of VPD occurring in the glasshouse despite cooling and water misting. Therefore, the weight of water transpired per leaf area, per day and per kPa of VPD (${{{{{{\rm{mg}}}}}}}_{{{{{{{\rm{H}}}}}}}_{2}0}$.cm^−2^.day^−1^. kPa_VPD_^−1^) was calculated.

Nitrogen acquisition and Symbiotic Nitrogen Fixation (SNF): To estimate N acquisition by plants, their leaf N concentration and an indicator of their SNF were determined. After drying and grinding (Retsch PM4 planetary grinder, Retsch, Haan, Germany), leaf N concentration was measured by an elemental analyser (NA 1500 NCS, Carlo Erba, Milan, Italy) for a subset of 112 samples encompassing all soil treatments and a wide range of plant size. Regarding SNF, most plants in this experiment did not initiate nodulation (because of toxicity and their small size). It was planned to use ^15^N soil labelling and the isotopic dilution method to estimate the efficiency of the SNF^65^, but it was not applicable in our experiment because of the low nodulation and the small amount of N derived from the atmosphere. Instead, at harvest, roots were cleaned gently and the number of nodules was determined.

### Statistics and reproducibility

All pots were placed randomly in the glasshouse at the beginning of the experiment. They were moved randomly every 15 days to avoid any spatial dependency between sample units. All statistical analyses were performed with R software (R Core Team, 2016). Regarding the biomass of plant parts, stems and leaves were considered together in a single shoot compartment when analysing the results. Bean stems are also photosynthetically active, and separate analyses for leaves gave consistent results.

All measured plant traits (SRA, SLA, water transpiration, nodule number, leaf N and chlorophyll concentration) can vary with plant ontogeny and plant size. Thus, variation of these traits (dependent variables) was analysed considering both plant size and soil treatments (explanatory variables). Shoot biomass was used as a surrogate for size for aboveground traits (SLA, water transpiration, leaf N, and chlorophyll concentration). Root biomass was used for belowground traits (root nodule number). Water transpiration was analysed by ANCOVA (shoot biomass as a regressor, soil treatment as a covariate). Leaf nitrogen and chlorophyll concentrations were first analysed by segmented linear modelling (segmented package) because of a radical change in the relationship between shoot biomass and these leaf traits at some size threshold. Then soil treatment effect on these traits was analysed on the residuals of the segmented relationships by ANOVA. Note that similar responses were observed for the different kinds of pigments, and only the results for chlorophyll_a+b_ concentrations are reported in this study. Similarly, we used a segmented linear model (segmented package) for the relationship between root nodule number and root biomass, and the soil treatment effect was analysed on this first model residuals. As to SLA and SRA, plants were grouped according to their shoot and root biomass tertile respectively. Then for each tertile, ANOVA was used to test the difference between SLA and SRA with soil treatment. When performing ANCOVAs, in the case of significant effects of soil treatment and interaction with plant size, post-hoc pairwise comparisons were used to test the difference of intercept or slopes between soil treatments (emmeans package). When performing ANOVAs, Post-hoc Tukey HSD pairwise comparisons were performed in case of significant effect of soil treatment. All variables were log-transformed when necessary to respect the condition of application of linear modelling.

When investigating allometric relationships between root and shoot biomass, or root and shoot areas, the interest is related to the analysis of how root biomass (or area) scales against shoot biomass (or area), rather than predicting the value of one variable from another. Standard Major Axis (SMA) regression (smatr package) on log-transformed variables was used accordingly to study this allometric scaling and its changes with soil treatments^66^. When changes in α scaling exponent with soil treatment are significant, estimation of differences in β (proportionality coefficient) between treatments is not enabled by SMA regression^66^. In that case, after estimating α with SMA regression, we estimated β value for each soil treatment using non-linear least square modelling (see Supplementary Table 2) because changes in β values have a biological meaning in our context (delay in early root development).

### Meta-analysis of literature dealing with plant biomass partitioning and modelling of changes in root: shoot ratio

Collection of published studies and case studies: we used the ISI Web of Science database to locate published studies on the effect of soil pollution on plant biomass partitioning. We entered a general query made using the combination of two phrases, one regarding biomass partitioning, and the other regarding soil pollution. We used several equivalent phrases regarding both terms, leading to the following query: (“biomass partitioning” OR “biomass partition” OR “biomass allocation” OR “root: shoot”) AND (“pollution” OR “contamination” OR “heavy metals” OR “PAH” OR “phytoremediation” OR “phytomanagement”). Some additional studies were picked out from the reference list found in the studies collected from our query. From the first selection of 53 potential studies (from their title and summary), the final collection made after careful reading of the entire studies comprised only 15 references (Table 2). From these studies, we identified 25 case studies suitable for the meta-analysis that was conducted in a large variety of geographical locations and climates. Studies and case studies were excluded from our database when M_R_: M_S_ could not be calculated, when they dealt with air pollution (not our subject), when no phytotoxic effects were shown (no decrease in plant growth), when no statistical analyses or tests had been done for the reported results regarding M_R_: M_S_ and root and shoot parts. When plant growth was reported both in hydroponic and for growth in soil substrates, we assumed that results from soil substrates where more suitable for analysing the biomass partitioning response. When other treatments were used (for instance mycorrhizae inoculation), we averaged the response to these treatments at each level of soil pollution. Finally, we considered one case study as being the unique combination of one team of researchers, one studied plant species, and one contaminant at stake. In one study, we made an exception and considered two case studies for two populations of the same plant species being exposed to the same contaminant, the two populations being reported as being metallicolous and non-metallicolous and which showed contrasting responses.

The statistics and information recorded: we aimed to answer three questions: Is there any general pattern (increase or decrease) of the M_R_: M_S_ ratio reported in the literature? Do changes in M_R_: M_S_ depend on some explicative factors (for instance the contaminant type)? and Do M_R_: M_S_ variations depend on pollution effect on plant size? This last question is important in our study which aimed to distinguish changes due to simple allometric effects rather than plant response. For each case study, the main indicator of biomass partitioning available was the M_R_: M_S_ ratio, either provided directly, or calculated from root and shoot biomasses. Total plant dry biomasses were also recorded. Then, we calculated two statistics to enable the comparison of studies that were not originally designed to be compared. Firstly, we calculated the effect size metric (referred to as relative response in this study) to estimate the effect of pollution on the M_R_: M_S_ ratio as follows:

3 $${{{Relative}}}\,{M}_{R}:{M}_{S}={{{{{\rm{log }}}}}}\big({M}_{R}:{M}_{S{{\_}}{{{polluted}}}}/{M}_{R}:{M}_{S{{\_}}{{{control}}}}\big)$$

Values close to 0 are associated with a negligible effect of the treatment, while negative and positive values indicate negative and positive effects of the treatment, respectively. The relative response is a reliable approach to quantify the effects of treatment compared to control and is regularly used in plant science (e.g. ref. ^67^). Secondly, we calculated a phytotoxic effect by normalisation of the effect of pollution on plant growth as follows:

4 $$Phytotoxicity\,(biomass\,loss)=1-\big({biomass}_{polluted}/{biomass}_{control}\big)$$

Values close to 0 are associated with a negligible effect on plant growth, while values close to 1 indicate a strong decrease in plant growth. Additionally, we report relevant information to analyse its potential influence on M_R_: M_S_ results. We reported the plant species involved, its functional group (monocotyledonous grass, dicotyledonous forb, and woody species), its life cycle (annual, perennial), the experiment duration (and if several measurements were made at different times) and the kind of contaminant at stake (see Table 2).

The meta-analysis: regarding the general pattern of M_R_: M_S_ changes_,_ they were classified on the basis of the statistical results reported in the different studies as follows: (i) stable: no change of the M_R_: M_S_ value was reported; (ii) variable: increase or decrease of the M_R_: M_S_ ratio was reported for a pollution treatment compared to the control, and other treatments with a higher level of pollution showed no or opposite effects; (iii) increase: increase of the M_R_: M_S_ ratio was reported for pollution treatment, and other treatments with higher levels of pollution also showed an increase compared to the control; (iv): decrease: an opposite situation to increase described above. Additionally, we tested the effect of the contaminant type, plant functional type, and plant life cycle on the relative M_R_: M_S_ by ANOVA. Finally, we tested the dependence of the relative M_R_: M_S_ with biomass loss for case studies showing an increase or decrease in this ratio by using linear modelling. This was done to compare results among case studies (one averaged value per case study). For results within a case study (when several soil pollution levels were available per case study), relative M_R_: M_S_ and biomass loss compared to the control was calculated for each pollution level. Rate of relative M_R_: M_S_ changes (Δ M_R_: M_S_ /Δ biomass loss) was calculated and compared to 0.

Modelling of changes in root: shoot ratio with exposure to pollution stress: we modelled the changes in the root: shoot ratio compared to a control situation without exposure to excess contaminants. We considered the change of allometric relationships (Eq. 1.) between root and shoot parts by the three potential drivers related to soil effect and plant response, and we followed the following steps. First, we calculated the growth of shoot parts as follows:

5 $$S={gr}\, .\,\left(1-{{gr}}_{{{{{{{\mathrm{decrease}}}}}}}}\right)\,.\,d$$

gr represents plant growth rate (it can concern shoot biomass or area) per day; d is the duration of the growing period (in days); gr_decrease_ is the phytotoxic effect on plant growth (interval [0,0.8] is considered here); S is the number of shoot parts produced after the corresponding duration d.

Second, we calculated corresponding root parts as follows

6 $$R={{\upbeta }}\,.\,\left(1-{{{\upbeta }}}_{{{{decrease}}}}\right)\,.\,{S}^{{{\upalpha }}.(1+{{\upalpha }}_{{{{increase}}}})}$$

With β and α the parameters of the allometric relationship of a given plant species in a control soil; β_decrease_ (the interval [0;0.5] is considered) is the effect of pollution stress on the early root development; α_increase_ (the interval [0;0.5] is considered) corresponds to plant response with increasing biomass partitioning in favour of roots; and R is the number of root parts produced.

Finally, changes in root: shoot ratios were calculated by dividing the root: shoot ratio obtained on polluted soils by the root: shoot ratio obtained in a control situation (gr_decrease_; β_decrease_; and α_increase_ set to 0).

### Reporting summary

Further information on research design is available in the Nature Research Reporting Summary linked to this article.

## Supplementary information

Supplementary Information

Reporting Summary

## Data Availability

Data are available from the Dryad digital repository^68^ 10.5061/dryad.44j0zpcgc
