# Supplementary material for: Biomass partitioning of plants under soil pollution stress
Source: Commun Biol. 2022 Apr 19;5:365. doi: 10.1038/s42003-022-03307-x (PMC9018880; doi:10.1038/s42003-022-03307-x)
Supplement: Supplementary file 1 — Supplementary Information [file 42003_2022_3307_MOESM1_ESM.pdf]

Authors : Delerue F[1,2*]., Scattolin M.[2], Atteia O.[1], Cohen G.[1], Franceschi M.[1], Mench M[3].

[1] Bordeaux INP, G&E, UMR 5805 EPOC, F-33600, Pessac, France

[2] Mines Saint-Etienne, Univ Lyon, Univ Jean Moulin, Univ Lumière, Univ Jean Monnet, ENTPE, INSA Lyon, ENS Lyon, CNRS, UMR 5600 EVS, Centre SPIN, Département PEG, F -42023 Saint-Etienne, France

[3] Univ. Bordeaux, INRAE, BIOGECO, F-33615 Pessac, France

*Author for correspondence: F. Delerue; Phone: +33 5 56 84 69 18; Fax: +33 56 84 69 01; Email: florian.delerue@bordeaux-inp.fr

Title

Biomass partitioning of plants under soil pollution stress

Supplementary Information:

Soil properties and contamination including **Supplementary Table 1**

Soil	pH_{H_20}	OM ($g.kg^{-1}$)	C_{ORG} ($g.kg^{-1}$)	N_{TOT} ($g.kg^{-1}$)	P_2O_5 Olsen ($mg.kg^{-1}$)	Cu_{TOT} ($mg.kg^{-1}$)	PAH ($mg.kg^{-1}$)
Control	5.47 ± 0.03 a	41.08 ± 0.20 b	23.77 ± 0.12 b	2.32 ± 0.02 c	10.7 ± 0.8 a	29.1 ± 6.9 a	49.8 ± 14.5 a
Cu-PAH	7.43 ± 0.02 c	14.92 ± 0.17 a	8.63 ± 0.10 a	0.57 ± 0.01 a	31.7 ± 0.6 b	888.9 ± 9.9 b	656.5 ± 311.4 b
HIGH Cu-PAH	5.85 ± 0.01 b	41.19 ± 0.71 b	23.79 ± 0.40 b	0.85 ± 0.01 b	44.8 ± 0.6 c	4276.0 ± 208.6 c	3141.8 ± 419.1 c

Supplementary Table 1. Soil C, N, and P concentrations and soil contaminants.

Mean and standard errors (from 10 samples analysed) are shown. The influence of soil origin on measured variables is significant in all cases ($P<0.001$, ANOVA). Significant differences between different soils are shown by different letters (post-hoc Tukey pairwise comparisons). OM (soil Organic Matter), C_{ORG} (soil Organic Carbon) and N_{TOT} (Total soil Nitrogen) were determined by dry combustion (NF ISO 10694). P_20_5 concentration was determined following the Olsen protocol (**NF ISO 11263)**, Cu_{TOT} (total soil Cu) was determined after solubilisation by HF and $HClO_4$ (NF X 31-147). PAH concentration refers to the sum of the 16 regulatory PAH (Office of the Federal Registration (OFR) 1982: Appendix A: priority pollutants. Fed Reg. 1982;47:52309). Soil PAH analyses were conducted following the protocol in[1].

A comment on the differences between the three soils used in this study:

Separating the responses to soil pollution from changes in other soil factors is often an intricate task[2]. Despite our care to use a control soil as similar as possible to the polluted soils, their distinct levels of pollution inevitably led to different chemical properties. The possibility has to be considered that an increase of biomass partitioning in favour of roots could also be due to the decrease of soil fertility for the polluted soils. Firstly, such an increase of root allocation due to lower soil fertility would be consistent with our theoretical framework presented in the introduction: pollution can decrease nutrient cycling and nutrient availability. In this context, the study of biomass allocation response is still relevant to highlight

the changes in ecosystem functioning. However, because we chose a leguminous species, differences in N nutrition are more due to a negative impact on the SNF than to the soil N availability. In addition, even if polluted soils were N-depleted compared to the control, their P availability was higher (Supplementary Table 1). Finally, even if we cannot exclude a response due to a change in soil fertility, the set of consistent results between specific root area, water transpiration and modification of allometric relationships described in the main text are also certainly under the influence of the effect of soil pollution on root morphology and of plant response to the decrease of resources capture.

Impact of soil treatments on leaves: complementary methods and results including **Supplementary Figure 1**.

Methods for estimating chlorophyll synthesis and photo-chemical energy:

Determination of the photosystem photo-chemistry efficiency by fluorescence can be used to assess the production of photo-chemical energy, but previous studies have indicated that it is not an early endpoint[3]. So, we assessed chlorophyll a, b and other carotenoid synthesis by determining their leaf concentrations. The day before harvesting, two 0.8 cm-diameter pieces of recently produced leaves were collected from the middle of each blade. They were placed in 3 mL of cooled (4°C) N-dimethylformamide (DMF) at 4°C for 48h. After extraction with DMF, Chl a, Chl b and total carotenoids in the extracts were measured spectrophotometrically (Varian Cary) at 470, 647 and 664.5 nm respectively, as described by Lichtenhaler and Wellburn[4].

Results and discussion:

Nitrogen acquisition and Symbiotic Nitrogen Fixation (SNF):

We found a strong relationship between leaf N concentrations and shoot biomass, from up to 7% of N for the smallest plant, to approximately 1.3% of N for plants with 0.72g shoot biomass (Supplementary Figure 1b). Then, an increase of leaf N concentration was observed for plants with higher shoot biomass, corresponding to plants in the control soil and in the 1/3 Cu-PAH soils, and at development stages 3 to 5 (Supplementary Figure 1b). Apart from this size dependent relationship, we did not observe any effect of soil treatment on leaf N concentration. Cotyledonous leaves were sampled for the smallest individuals. Thus, a simple interpretation of our results is a decrease in leaf N concentration because of the dilution of the initial N stock in green shoots of cotyledons and a decrease of leaf C:N ratio as plants grow (e.g.[5]). Additionally, the nodulation and SNF observed for bigger plants in the control soil and the 1/3 Cu-PAH soil may explain their increase of N concentration in leaves.

Light and photo-chemical energy

Similar responses were observed for the different kinds of pigments, but only the results for $chlorophyll_{a+b}$ concentrations are reported in this study. $Chlorophyll_{a+b}$ concentration showed a similar, but less marked pattern than that of leaf N concentration (Supplementary Figure 1c). We found some deviations from this global relationship, with plants growing on the 2/3 HIGH Cu-PAH soil having lower concentrations (Supplementary Figure 1d). However, the different soil treatments did not appear to impact chlorophyll content consistently with soil dilution and

contamination. These results are in line with the strong correlation between leaf N and Chlorophyll concentrations[6]. Apart from this change with plant size, our results did not suggest a quantitative impact of the pollution gradients on photosystem synthesis. Nonetheless, because we did not investigate the photo-chemistry efficiency by fluorescence (e.g.[7]), we cannot strictly conclude that soil pollution did not modify the ability of plants to capture light.

Figure

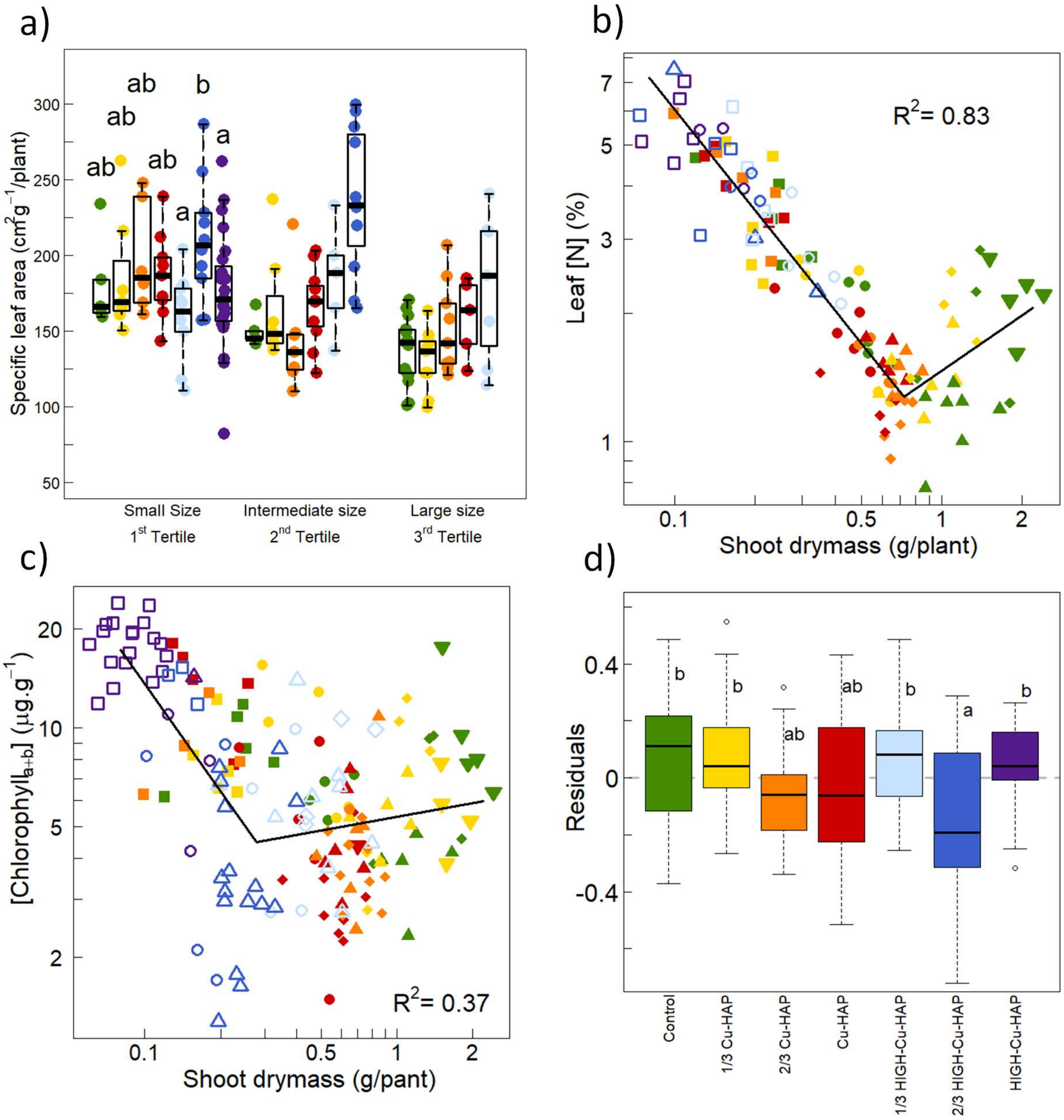

Supplementary Figure 1: Impact of soil treatments on leaves.

Symbols and colors are as in Figure 1 in the main text.

a) Modification of Specific Leaf Areas (SLA) according to soil treatments. All plants were split into three groups according to shoot biomass tertiles to be consistent with Fig 1a. Because of the negative effect on plant growth (Table 1), plants from the highest phytotoxic soils (2/3 HIGH-Cu-PAH and HIGH-Cu-PAH) are not represented in the highest size class (third tertile), and plants from the HIGH-Cu-PAH soil are not represented in the intermediate size class (second tertile). ANOVA were performed to detect differences of SLA with soil treatments. Different letters indicate significant differences between soil treatments (post-hoc Tukey pairwise comparisons).

b) Size-dependent relationship between shoot biomass and leaf N concentration. The segmented regression shown is highly significant ($P < 0.001$ for the existence of a breakpoint, Davies test) indicating two different relationships, a negative one when shoot biomass is inferior to 0.72g, and then a positive one. The soil treatment has no effect on this segmented relationship.

c) Size-dependent relationship between shoot biomass and leaf Chl_{a+b} concentration. The segmented regression shown is highly significant ($P < 0.001$ for the existence of a breakpoint, Davies test) indicating two different relationships, a negative one when shoot biomass is inferior to 0.27 g, and then a positive one.

d) Residuals of the segmented relationship between shoot biomass and leaf Chlorophyll a+b concentration (panel c). Soil treatment being significant ($P<0.001$, ANOVA), different letters indicate a significant difference between groups (post-hoc Tukey pairwise comparisons).

Impact of soil treatment on the variation of root mass and area fractions with plant size including **Supplementary Figure 2**

In addition to allometric relationship analysis (Fig 2 in the main text), it has been suggested to present complementary analysis of environmental influence on the organ mass fraction variation with plant size[8]. Organ Mass Fractions (OMF) are calculated as follow:

$$OMF = \frac{Organ\ Mass}{Total\ Plant\ Mass} \qquad (S1)$$

These fractions provide a straightforward biological interpretation of biomass partitioning because they are readily understandable. Here, as for allometric relationships, we also analysed partitioning to root and shoot in terms of resource acquisition surfaces. Thus, we also calculated Organ Area Fractions (OAF) as follow:

$$OAF = \frac{Organ\ Area}{Total\ Plant\ Area} \qquad (S2)$$

In this study, whole plants were split in only two "organs" (roots and shoots). Thus results of one organ fraction (e.g. roots) is fully complementary of results for the other organ fraction (e.g. shoots). Because our results and interpretations focus on biomass allocation to roots, we present results of root fractions (Supplementary Figure 2). As to statistics, ANCOVAs were performed to test the relationship between fractions (dependent variable) and plant size, soil treatment and their interaction. Plant total biomass was used as a surrogate for plant size when analysing Root Mass Fractions; plant total area was used as a surrogate for plant size when analysing Root Area Fractions.

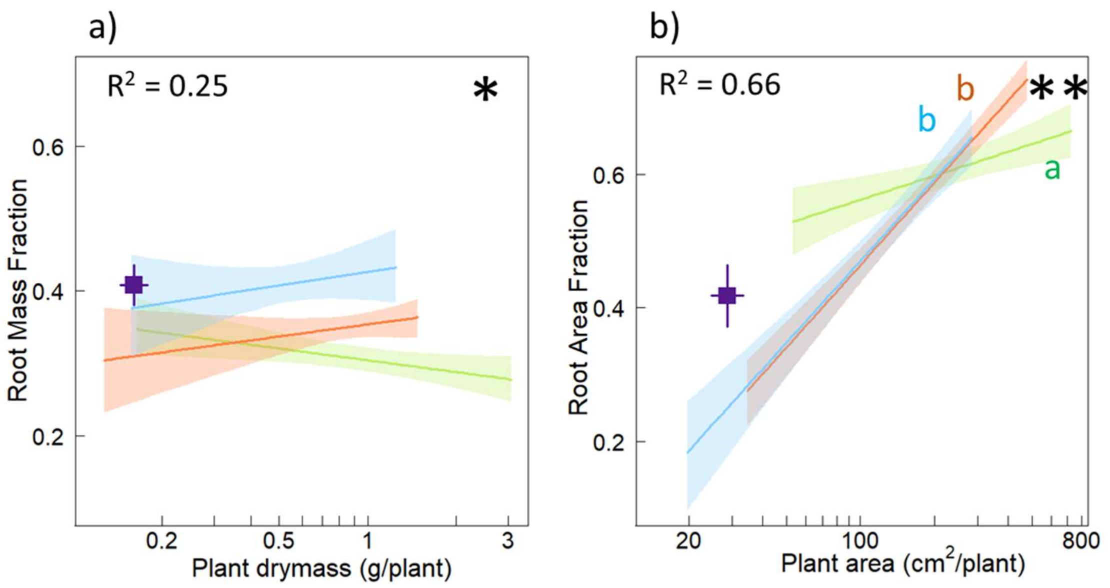

Supplementary Figure 2. Variation of Root Mass (a) and Area (b) Fractions according to plant size and soil treatment.

For the sake of clarity, relationships for the different soil treatments are presented in three groups as in Fig 2 (Control and 1/3 Cu-PAH soils in green; 2/3 Cu-PAH and Cu-PAH soils in red; 1/3 HIGH-Cu-PAH and 2/3 HIGH-Cu-PAH soils in blue). Fitted relationships (lines) and 95% confidence envelopes (shaded areas) are shown except for plants from the HIGH-Cu-PAH soil (see the dark purple square for HIGH-Cu-PAH mean ± SE), because they did not grow. R^2 are shown on the top-left corner. The significance level of the interaction between plant size and soil treatment is shown on the top-right of the panels (*; $P<0.05$; **; $P<0.01$). Lowercase letters indicate a difference of slope between groups (post-hoc pairwise comparisons; $P<0.05$). For Root Mass Fractions, the interaction (plant size x soil treatment) is significant, but adjustment of p-values for multiple pairwise comparisons led to no significant difference within each pair of groups.

Plant size (mass or area) was log-transformed. The interaction between plant-size and soil treatment being significant in all case, it does not enable a straightforward interpretation of intercept differences (at $\log_{10}X = 0$; equivalent to $X = 1$) because it depends on the unit use for roots and shoots (for instance g, cg, mg for mass; cm^2, mm^2 for area). Difference of fractions for small plants are better highlighted by the absence of overlap between confidence envelopes. While important differences of Root Area Fractions appear for small plants in polluted soils compared to the control, these differences are not visible when considering Root Mass Fractions.

a) Root and shoot mass analysis (Standard Major Axis regression)			
With all soil treatments (Fig.2a)	$Root_{mass\_log} \sim Shoot_{mass\text{-}log}$*Soil		
Soil	slope (α) ***		$CI_{95\%}$ of $\beta^{\$}$
Control	0.92	a	0.38-0.48
1/3 Cu-PAH	0.93	a	0.46-0.58
2/3 Cu-PAH	1.24	ab	0.32-0.40
Cu-PAH	1.02	ab	0.46-0.55
1/3 HIGH Cu-PAH	1.49	b	0.22-0.30
2/3 HIGH Cu-PAH	1.31	ab	0.50-0.68
HIGH Cu-PAH#	1.05	ab	0.56-0.72
Simplified analysis (Fig. 2a, bottomright)			
Soil	slope (α)*		$CI_{95\%}$ of $\beta^{\$}$
Control - 1/3 Cu-PAH	0.92	a	0.43 - 0.51
2/3 Cu-PAH / Cu-PAH	1.15	b	0.39 - 0.45
1/3 HIGH Cu-PAH - 2/3 HIGH Cu-PAH	1.19	b	0.43 - 0.54

b) Root and shoot area analysis (Standard Major Axis regression)			
With all soil treatments (Fig.2b)	$root_{area\_log} \sim shoot_{area\text{-}log}$*Soil		
Soil	slope (α)***		$CI_{95\%}$ of $\beta^{\$}$
Control	1.01	a	1.126-1.583
1/3 Cu-PAH	1.40	ab	0.184-0.263
2/3 Cu-PAH	1.93	bc	0.016-0.027
Cu-PAH	2.25	c	0.004-0.006
1/3 HIGH Cu-PAH	2.34	c	0.002-0.004
2/3 HIGH Cu-PAH	1.95	bc	0.017-0.027
HIGH Cu-PAH#	1.27	abc	0.204-0.355
Simplified analysis (Fig. 2b, bottomright)			
Soil	slope (α)***		$CI_{95\%}$ of $\beta^{\$}$
Control - 1/3 Cu-PAH	1.21	a	0.432-0.566
2/3 Cu-PAH / Cu-PAH	1.93	ab	0.016-0.027
1/3 HIGH Cu-PAH - 2/3 HIGH Cu-PAH	2.12	b	0.008-0.010

Supplementary Table 2. Detailed results of statistical models corresponding to Figure 2.

Each part of the table (a, b) corresponds to the same panel (a, b) in Figure 2. Two results are shown. Firstly, results from a standard major axis regression are shown which consider all soil treatments. Secondly, results from a standard major axis regression are shown with a simplification of soil treatments in three groups as in Fig. 2. Performing standard major axis regressions, overall significance of soil treatments on slope (α coefficient) are shown. Different letters indicate significant differences between groups ($P<0.05$, pairwise multiple comparisons) (***, $P < 0.001$; **, $P<0.01$; * $P<0.05$).

$: Performing SMA regression in the log-log scale, test of intercept term (log β, see equation 2 in the main text) differences between treatments is not possible when slopes of the different treatments are different as here. Additionally, the unit use for roots and shoots (for instance g, cg, mg

for mass; cm^2, mm^2 for area) will dramatically change the values of log β and statistical results. Instead, once values of α had been determined by SMA regression, we used non-linear least square regression to fit the relationships into natural scale (see equation 1) to determine values of β and their 95% confidence interval for each soil treatment as reported here. Differences of β shown in Fig.2 indicate that these confidence intervals do not overlap.

Figure and panel	Dependent variable	Factor	Df	Test statistic		p-value
1a. First tertile	Specific Root Area	Soil treatment	6	15.11	F	< 0.001
1a. Second tertile	Specific Root Area	Soil treatment	5	11.86	F	< 0.001
1a. Third tertile	Specific Root Area	Soil treatment	4	9.52	F	< 0.001
1b	Water transpiration	Shoot biomass	1	401.38	F	< 0.001
		Soil treatment	6	24.04	F	< 0.001
		Shoot biomass x Soil treatment	6	2.12	F	0.053
1c - segmented relationship	slope before breakpoint	Root biomass	4$	-3.10^{-15}	t	> 0.10
	slope after breakpoint	Root biomass	4$	5.24	t	< 0.01
1c - residuals	residuals	Soil treatment	35	2.94	t	< 0.01
2a	test of common slopes (α)#	soil treatment	5	17.84	LR	< 0.01
2b	test of common slopes (α)#	soil treatment	5	38.04	LR	< 0.01
3a	Relative M_R: M_S (increase class)	Biomass loss	1	9.53	F	< 0.05
3a	Relative M_R: M_S (decrease class)	Biomass loss	1	1.83	F	> 0.10
3a	Relative M_R: M_S (decrease class)	Fonctionnal type	2	4.83	F	< 0.05

Supplementary Table 3: Degrees of freedom, statistical tests and corresponding p-values for the main statistical models of Figures 1, 2 and 3.

$: Test statistics and p-values are provided when performing segmented regression with the "segmented" package. Calculation of degrees of freedom are not indicated, but as we know the distribution of the test statistic (t is for Student), we estimated them from a Student distribution, the statistic value and corresponding p-value.

#: Standard major axis regression does not consider dependent or independent variables like in ordinary least square regression. Still, the statistical test performed checked the difference of slopes (α scaling coefficients) between groups.

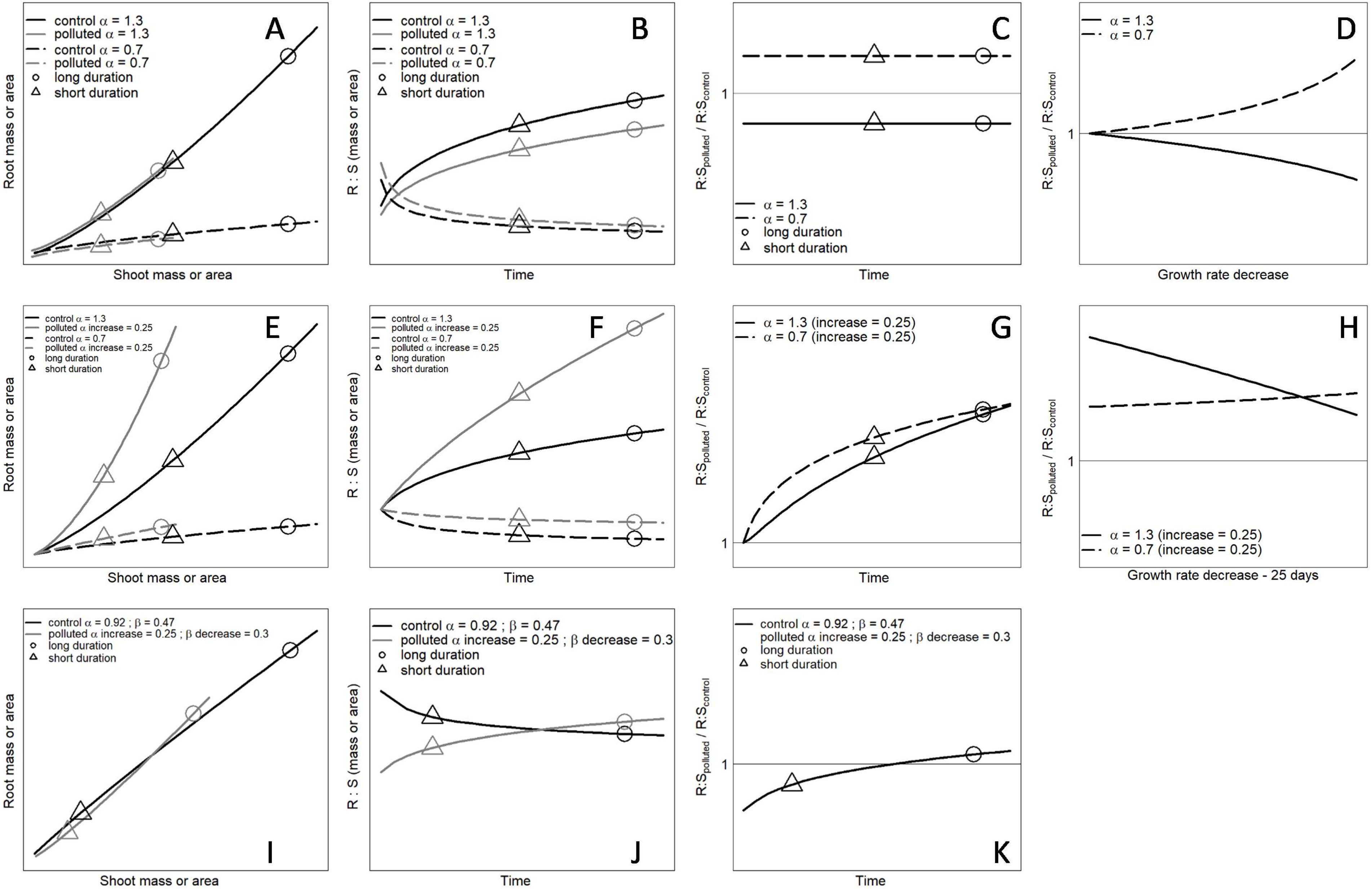
A
control α = 1.3
polluted α = 1.3
control α = 0.7
polluted α = 0.7
long duration
short duration
Root mass or area
Shoot mass or area
B
control α = 1.3
polluted α = 1.3
control α = 0.7
polluted α = 0.7
long duration
short duration
R : S (mass or area)
Time
C
1
α = 1.3
α = 0.7
long duration
short duration
R:Spolluted / R:Scontrol
Time
D
α = 1.3
α = 0.7
1
R:Spolluted / R:Scontrol
Growth rate decrease
E
control α = 1.3
polluted α increase = 0.25
control α = 0.7
polluted α increase = 0.25
long duration
short duration
Root mass or area
Shoot mass or area
F
control α = 1.3
polluted α increase = 0.25
control α = 0.7
polluted α increase = 0.25
long duration
short duration
R : S (mass or area)
Time
G
α = 1.3 (increase = 0.25)
α = 0.7 (increase = 0.25)
1
R:Spolluted / R:Scontrol
Time
H
1
α = 1.3 (increase = 0.25)
α = 0.7 (increase = 0.25)
R:Spolluted / R:Scontrol
Growth rate decrease - 25 days
I
control α = 0.92 ; β = 0.47
polluted α increase = 0.25 ; β decrease = 0.3
long duration
short duration
Root mass or area
Shoot mass or area
J
control α = 0.92 ; β = 0.47
polluted α increase = 0.25 ; β decrease = 0.3
long duration
short duration
R : S (mass or area)
Time
K
control α = 0.92 ; β = 0.47
polluted α increase = 0.25 ; β decrease = 0.3
long duration
short duration
1
R:Spolluted / R:Scontrol
Time

Supplementary Figure 3: Some examples of root: shoot changes with soil pollution according to the three scenarios presented in Fig.4

For all examples, we show the growth trajectory (roots with regard to shoots) in left-hand panels (A, E, I), and we positioned two observations: one after a short (triangle) period of growth and a second after a longer duration (circle). Corresponding evolution of root: shoot ratios with time are shown in panels B, F and J. Modifications of root: shoot ratios on polluted soils compared to a control situation are shown in panel C, G, H. Modifications of root: shoot ratios on polluted soils compared to the control are shown with regard to the impact of pollution on plant growth (toxic effect) in panels D and H. α increases and β decreases indicated in this figure are those considered in Eq. 6 in the main text. Daily shoot growth rate for the control situation was set to 5 $unit.day^{-1}$ in all simulations, and the number of days of growth varies between 1 and 50. Note that different growth rates and durations will produce the same patterns, but after different growing durations.

A, B, C, D): the first case in Figure 4 (change in growth rate only). Because of a growth reduction (toxic effect), plants in polluted soils have either lower or higher root: shoot ratios when they show an $\alpha > 1$ or $\alpha < 1$ respectively (B, C). See also Fig. 4b. This change in root: shoot ratio is independent of time (C), and only depends on the strength of growth rate decrease (D, toxic effect), leading to patterns similar to those found in the literature (Fig. 3a, b).

E, F, G, H): Second case in Figure 4 (change in growth rate and allometric response (α increase)). The allometric increase of root allocation (plant response) leads to higher root: shoot ratios in any case on polluted soils (F), and this increase is stronger with time (G). At a given time, this increase is also related to growth rate reduction (H). When plants on polluted soils have an $\alpha > 1$ (after considering the allometric change in response to pollution), a higher growth rate decrease leads to a less important increase of root: shoot ratios. Conversely, when plants on polluted soils still have an $\alpha < 1$ (after considering the allometric change in response to pollution), a higher growth rate decrease leads to greater increase of root: shoot ratios (H). In sum, the increased root: shoot ratio on polluted soils is ubiquitous in this situation. Its intensity depends on both growth rate decrease and time.

I, J, K) Third case in Figure 4 (change in growth rate, impact on early development (β decrease) and allometric response (α increase)). Here, we used α and β values for the control corresponding to this study (allometric relationship between root and shoot biomasses). The initial delay in root development (β decrease) is compensated for by higher root allocation (α increase) for bigger plants. This leads to a possible change in root: shoot ratio response to pollution with time (from lower values to higher values on polluted soils (J, K)). Variation of the intensity of growth rate decrease (not shown here) will produce the same patterns, but after different durations. In sum, the change in root: shoot ratios on polluted soils can have different directions (increase and decrease). For a given change in allometric relationship (a combination of α increase and β decrease), the final results will depend on both growth rate decrease and time (see also Fig. 4c,d).

Modelling approach with modification of the scaling exponent with plant growth including **Supplementary Figure 4**

The large majority of published literature regarding within species allometric relationships show linear log-log relationship with unvarying α scaling coefficient with plant size. But some exceptions have been reported. These nonlinear log-log relationships can be better modelled by quadratic polynomial regression (see Fig 2 in [8]). This correspond to the following equation:

$$\log (Y) = \log (\beta) + \alpha \log (X) + \delta \log (X)^2 \quad \text{(S3)}$$

Note that in our case study (Fig 2), quadratic polynomial regressions were not significant (δ not different from 0, P=0.16 and P=0.48 for mass and area relationships respectively); thus log-log relationships were linear.

Considering Eq. S3, the scaling term between x and y (the slope of the relationship) vary with X and is equal to ($\alpha + \delta \log(X)$), the derivative of Eq. S3.

Additionally, equation S3 is equivalent to:

$$Y = \beta.X^{(\alpha + \delta.\log X)} \text{ or in our case } R = \beta.S^{(\alpha + \delta.\log S)} \quad \text{(S4)}$$

With R and S corresponding to root and shoot parts respectively. Then, from Equation S4, the modification of the R: S ratio with soil pollution and in comparison with a control situation can follow the same procedure stated in the main text. We considered the change of R: S by the three potential drivers related to soil effect and plant response with the following steps.

First, we calculated the growth of shoot parts as in the main text:

$$S = gr \,.\, (1-gr_{decrease}) \,.\, d \quad (5)$$

gr represents plant growth rate (it can concern shoot biomass or area) per day; d is the duration of the growing period (in days); $gr_{decrease}$ is the phytotoxic effect on plant growth (interval [0,0.8] is considered here); S is the amount of shoot parts produced after the corresponding duration d.

Second, we calculated corresponding root parts as follow

$$R = \beta.(1-\beta_{decrease}) \,.\, S^{(\alpha + \delta.\log S).(1+\text{scaling increase})} \quad \text{(S5)}$$

With β, α and δ the parameters of the relationship of a given plant species in a control soil; $\beta_{decrease}$ (the interval [0;0.5] is considered) is the effect of pollution stress on the early root development; *scaling increase* (the interval [0;0.5] is considered) corresponds to plant response with increasing biomass partitioning in favour of roots; and R is the amount of root parts produced. Equation S5 is similar to Equation 6 in the main text. The increase of biomass partitioning (here *scaling increase*) apply not only to α but to the all scaling term which is now equal to ($\alpha + \delta.\log S$).

Finally, changes in R: S ratios were calculated by dividing the R: S ratio obtained on polluted soils by the R: S ratio obtained in a control situation ($gr_{decrease}$; $\beta_{decrease}$ and $_{scaling\ increase}$ set to 0).

The following figure (Supplementary Figure 4) shows the main results of this modelling approach.

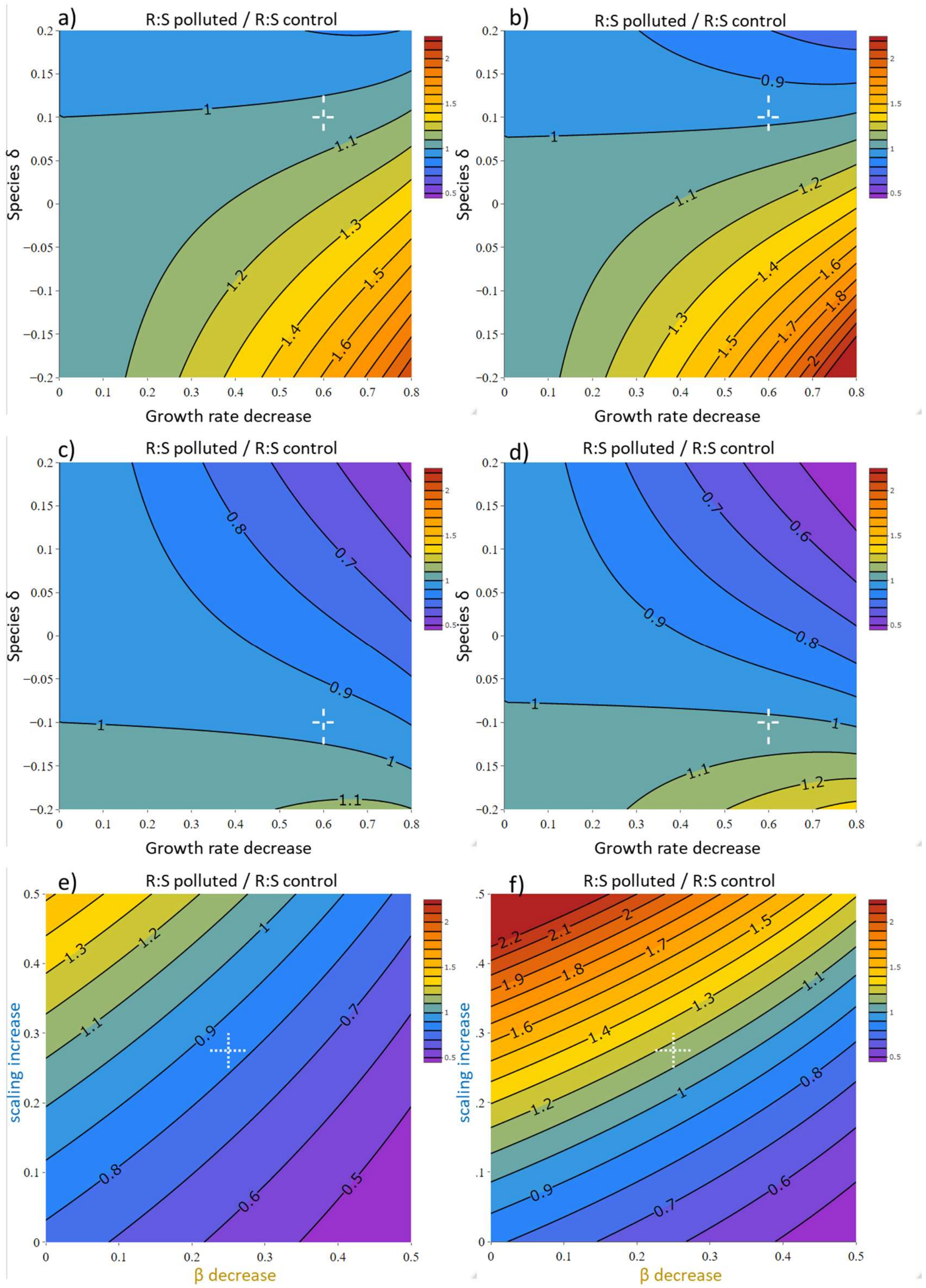

Supplementary Figure 4: Modelled changes in R: S ratio compared to a control situation (growth in unpolluted soil) considering a change in the scaling between root and shoot as plants grow.

Increase and decrease rates are indicated on the different axes (see equations 5 and S5). Parameters used for the simulations were as follows.

Daily shoot growth rate for the control situation: 5 unit.day^{-1} in all cases.

In a-d): β for control situation: 0.47 (this study). The scaling between root and shoot depend on (α + δ.logS). δ varied within [-0.2; 0.2]. In a,b) α is set to 0.8. In c,d) α is set to 1.2. In a,c) Number of day of growth: 20. In b,d) Number of day of growth: 40.

In e and f) Growth rate decrease: 0.5. To set complementary value of α and δ, we used the values corresponding to the relationship fitted in our case study in Fig 2b (α = 1.56; δ = -0.16), event if δ was not significant. Number of day of growth: 10 in c) and 20 in d)

Note that different parameter values will produce the same patterns but after different growing durations.

a-d) Changes in R:S in the absence of plasticity (**Case 1** in Fig 4a) according to the species scaling term (α + δ.logS) and elapsed time.

Difference between a) and b) and between c) and d) are due to elapsed time (20 days in a,c) and 40 days in b,d)). White crosses position the same changes of δ and growth rate decrease.

Difference between a) and c) and between b) and d) are due to differences in α (0.8 in a,c) and 1.2 in b,d)).

e and f) a decrease of early root development is considered (β decrease) for plants exposed to soil pollution, and then a plastic increase of biomass allocation (scaling increase) occurs to offset the decrease in soil resource acquisition (Fig 2b in this study for root and shoot areas, **Case 3** in Fig 4a in the main text). White crosses position the same changes of β and scaling increase.

Note that in case of a plastic increase of biomass partitioning (scaling increase) involved in response to pollution stress while no β decreased is observed (**Case 2** in Fig 4a in the main text) R:S will be higher for plants exposed to pollution.

We considered three scenarios of increasing complexity to scrutinize potential changes in root: shoot ratio as in Figure 4a. The simplest scenario implied a decrease of plant growth rate (toxic effect; Supplementary Figure 4a-d). When species α increased (comparison of Supplementary Figure 4a and and 4c and of Supplementary Figure 4b and 4d) R: S tended to decrease with pollution in relation with growth rate reduction as shown in the main text (Fig 4b). δ modifications exacerbated this effect. The more δ increased, the more R: S with soil pollution decreased and reversely (Supplementary Figure 4a-d). Note that because of the nonlinear log-log relationship and variation of the scaling term as plants grow, results for R: S were influenced by the elapsed time. The same set of parameter values (β, α, δ, growth rate decrease) can lead to either increase or decrease of R: S depending of the elapsed

time (comparison of Supplementary Figure 4a and 4b and of Supplementary Figure 4c and 4d, see the white crosses).

The second scenario required a growth rate decrease (toxic effect) and a plastic increase of the scaling term (plant response). It will lead to an increase of root: shoot ratio in all cases, either the log-log relationship is quadratic (Eq. S3) or linear (Eq 2).

The third scenario involved a growth rate decrease (toxic effect), a plastic increase of the scaling term ($\alpha + \delta$.logS; plant response) and a decrease of β (toxic effect on initial root development). As in Figure 4c,d in the main text, the net effect on the root: shoot ratio was complex and depended on the relative importance of changes in the scaling term and β, and on the duration of the growth period (Supplementary Figure 4e,f). An important β decrease could be compensated for by an important scaling term increase, or by a moderate scaling term increase and a longer period of growth. Noticeably, with the same changes of growth rate, scaling term and β, the change in root: shoot ratio could vary with elapsed time, being decreased, unchanged or increased compared to the control (Supplementary Figure 4e,f, see the white crosses).

To sum up, the consideration of nonlinear log-log relationships added some complexity to the modelling approach because of the need to consider different combinations of α and δ to define the scaling term. However, the main trends and interpretations proposed in the main text remained unchanged. Different possible combinations of the three drivers of biomass partitioning (growth rate decrease, β decrease, scaling term increase) can lead to the same R: S ratio change. Additionally, the same combination of the three drivers can lead to different R: S changes with elapsed time variations. Even when considering the simplest scenario (only one driver involved, the toxic reduction of growth rate), R: S changes depended on elapsed time variations. The modelling approach was based first on the estimation of plant development trajectory in a control situation. Then, in a second step, deviation from this trajectory due to toxic effects or plant response are considered (through Eq. S5). Thus, the form of the trajectory of plant development in the control situation, whether it fits a linear log-log relationship or not, has little influence on the conclusion provided by the modelled trends of the R: S ratio changes.

Supplementary References (for the references included in the meta-analysis, see below)

1. Mahamat-Ahmat, A., Cohen, G. & Atteia, O. The influence of soil mechanical redesigning on Polycyclic Aromatic Hydrocarbon (PAH) release: a column approach. *Water, Air, & Soil Pollution* **230**, 148 (2019).
2. Delerue, F., Masfaraud, J.-F., Lascourrèges, J.-F. & Atteia, O. A multi-site approach to investigate the role of toxicity and confounding factors on plant bioassay results. *Chemosphere* **219**, 482–492 (2019)
3. Marchand, L., Lamy, P., Bert, V., Quintela-Sabaris, C. & Mench, M. Potential of *Ranunculus acris* L. for biomonitoring trace element contamination of riverbank soils: photosystem II activity and phenotypic responses for two soil series. *Environmental Science and Pollution Research* **23**, 3104–3119 (2016).
4. Lichtenthaler, H. K. & Wellburn, A. R. Determinations of total carotenoids and chlorophylls a and b of leaf extracts in different solvents. *Biochemical Society Transactions* **11**, 591–592 (1983).
5. Coleman, J. S., McConnaughay, K. D. M. & Bazzaz, F. A. Elevated CO_2 and plant nitrogen-use: is reduced tissue nitrogen concentration size-dependent? *Oecologia* **93**, 195–200 (1993).
6. Evans, J. R. Photosynthesis and nitrogen relationships in leaves of C3 plants. *Oecologia* **78**, 9–19 (1989).
7. Paunov, M., Koleva, L., Vassilev, A., Vangronsveld, J. & Goltsev, V. Effects of different metals on photosynthesis: Cadmium and Zinc affect chlorophyll fluorescence in durum wheat. *International Journal of Molecular Sciences* **19**, 787 (2018).
8. Poorter, H. & Sack, L. Pitfalls and possibilities in the analysis of biomass allocation patterns in plants. *Frontiers in Plant Science* **3**, 259 (2012).

References included in the meta-analysis (and numbered as in Table 2).

1. Cai, X. *et al.* Biomass allocation strategies and Pb-enrichment characteristics of six dwarf bamboos under soil Pb stress. *Ecotoxicology and Environmental Safety* **207**, 111500 (2021).
2. Liu, J., Xiong, Z., Li, T. & Huang, H. Bioaccumulation and ecophysiological responses to copper stress in two populations of *Rumex dentatus* L. from Cu contaminated and non-contaminated sites. *Environmental and Experimental Botany* **52**, 43–51 (2004).

3. Merkl, N., Schultze-Kraft, R. & Infante, C. Assessment of tropical grasses and legumes for phytoremediation of petroleum-contaminated soils. *Water, Air, and Soil Pollution* **165**, 195–209 (2005).
4. Nie, M. *et al.* Do plants modulate biomass allocation in response to petroleum pollution? *Biology Letters* **6**, 811–814 (2010).
5. Iori, V. *et al.* Growth responses, metal accumulation and phytoremoval capability in *Amaranthus* plants exposed to nickel under hydroponics. *Water, Air, & Soil Pollution* (2013) doi:10.1007/s11270-013-1450-3.
6. Xiong, Z.-T., Liu, C. & Geng, B. Phytotoxic effects of copper on nitrogen metabolism and plant growth in *Brassica pekinensis* Rupr. *Ecotoxicology and Environmental Safety* **64**, 273–280 (2006).
7. Zhang, L., Pan, Y., Lv, W. & Xiong, Z. Physiological responses of biomass allocation, root architecture, and invertase activity to copper stress in young seedlings from two populations of *Kummerowia stipulacea* (maxim.) Makino. *Ecotoxicology and Environmental Safety* **104**, 278–284 (2014).
8. Uveges, J. L., Corbett, A. L. & Mal, T. K. Effects of lead contamination on the growth of *Lythrum salicaria* (purple loosestrife). *Environmental Pollution* **120**, 319–323 (2002).
9. Brennan, A., Jiménez, E. M., Puschenreiter, M., Alburquerque, J. A. & Switzer, C. Effects of biochar amendment on root traits and contaminant availability of maize plants in a copper and arsenic impacted soil. *Plant and Soil* **379**, 351–360 (2014).
10. Danh, L. T., Truong, P., Mammucari, R. & Fostert, N. Economic incentive for applying vetiver grass to remediate lead, copper and zinc contaminated soils. *International Journal of Phytoremediation* **13**, 47–60 (2011).
11. Haroni, N. N., Badehian, Z., Zarafshar, M. & Bazot, S. The effect of oil sludge contamination on morphological and physiological characteristics of some tree species. *Ecotoxicology* **28**, 507–519 (2019).

12. Nakata, C., Qualizza, C., MacKinnon, M. & Renault, S. Growth and physiological responses of *Triticum aestivum* and *Deschampsia caespitosa* exposed to petroleum coke. *Water, Air, and Soil Pollution* **216**, 59–72 (2011).

13. Noori, A. S., Maivan, H. Z. & Alaie, E. *Leucanthemum vulgare* lam. germination, growth and mycorrhizal symbiosis under crude oil contamination. *International Journal of Phytoremediation* **16**, 962–970 (2014).

14. Ryser, P. & Emerson, P. Growth, root and leaf structure, and biomass allocation in *Leucanthemum vulgare* Lam. (Asteraceae) as influenced by heavy-metal-containing slag. *Plant Soil* **301**, 315–324 (2007).

15. Vondráčková, S., Tlustoš, P. & Száková, J. Can liming change root anatomy, biomass allocation and trace element distribution among plant parts of *Salix* × *smithiana* in trace element-polluted soils? *Environmental Science and Pollution Research International* **24**, 19201–19210 (2017).
