# Supplementary material for: Biomass partitioning of plants under soil pollution stress
Source: Commun Biol. 2022 Apr 19;5:365. doi: 10.1038/s42003-022-03307-x (PMC9018880; doi:10.1038/s42003-022-03307-x)
Supplement: Supplementary file 2 — Reporting Summary [file 42003_2022_3307_MOESM2_ESM.pdf]

nature portfolio

Corresponding author(s): Florian DELERUE

Last updated by author(s): Feb 21, 2022

Reporting Summary

Nature Portfolio wishes to improve the reproducibility of the work that we publish. This form provides structure for consistency and transparency in reporting. For further information on Nature Portfolio policies, see our Editorial Policies and the Editorial Policy Checklist.

Statistics

For all statistical analyses, confirm that the following items are present in the figure legend, table legend, main text, or Methods section.

n/a	Confirmed	
☐	☒	The exact sample size (n) for each experimental group/condition, given as a discrete number and unit of measurement
☐	☒	A statement on whether measurements were taken from distinct samples or whether the same sample was measured repeatedly
☐	☒	The statistical test(s) used AND whether they are one- or two-sided<br>*Only common tests should be described solely by name; describe more complex techniques in the Methods section.*
☐	☒	A description of all covariates tested
☐	☒	A description of any assumptions or corrections, such as tests of normality and adjustment for multiple comparisons
☐	☒	A full description of the statistical parameters including central tendency (e.g. means) or other basic estimates (e.g. regression coefficient) AND variation (e.g. standard deviation) or associated estimates of uncertainty (e.g. confidence intervals)
☐	☒	For null hypothesis testing, the test statistic (e.g. F, t, r) with confidence intervals, effect sizes, degrees of freedom and P value noted<br>*Give P values as exact values whenever suitable.*
☒	☐	For Bayesian analysis, information on the choice of priors and Markov chain Monte Carlo settings
☒	☐	For hierarchical and complex designs, identification of the appropriate level for tests and full reporting of outcomes
☐	☒	Estimates of effect sizes (e.g. Cohen's d, Pearson's r), indicating how they were calculated

Our web collection on statistics for biologists contains articles on many of the points above.

Software and code

Policy information about availability of computer code

Data collection	*Provide a description of all commercial, open source and custom code used to collect the data in this study, specifying the version used OR state that no software was used.*
Data analysis	*Provide a description of all commercial, open source and custom code used to analyse the data in this study, specifying the version used OR state that no software was used.*

For manuscripts utilizing custom algorithms or software that are central to the research but not yet described in published literature, software must be made available to editors and reviewers. We strongly encourage code deposition in a community repository (e.g. GitHub). See the Nature Portfolio guidelines for submitting code & software for further information.

Data

Policy information about availability of data

All manuscripts must include a data availability statement. This statement should provide the following information, where applicable:

- Accession codes, unique identifiers, or web links for publicly available datasets
- A description of any restrictions on data availability
- For clinical datasets or third party data, please ensure that the statement adheres to our policy

Data available from the Dryad digital repository https://doi.org/10.5061/dryad.44j0zpcgc

Field-specific reporting

Please select the one below that is the best fit for your research. If you are not sure, read the appropriate sections before making your selection.

☐ Life sciences ☐ Behavioural & social sciences ☒ Ecological, evolutionary & environmental sciences

For a reference copy of the document with all sections, see nature.com/documents/nr-reporting-summary-flat.pdf

Ecological, evolutionary & environmental sciences study design

All studies must disclose on these points even when the disclosure is negative.

Study description

This study aimed at: (i) having an empirical evaluation of the different drivers of biomass partitioning with soil pollution stress; and (ii) performing an in-depth literature survey to verify the ability of former studies to distinguish between the various drivers under investigation. The details provided here and in response to the other questions below concern the empirical part of this study.
We created two soil series with increasing Cu and PAH contamination by diluting two contaminated soils with an uncontaminated control soil harvested nearby, with a similar soil texture. The addition rates were 0%, 33%, 66%, and 100% of contaminated soil to the control soil for each soil series given a total of 7 different soils. Dwarf bean plants were cultivated on these soils (one plant per pot) in a glasshouse, and harvested at 5 development stages (from the end of cotyledon opening-stage 1 to 6 trifoliate leaves-stage 5) to ensure a wide range of plant size in the dataset. Five dwarf bean plants were cultivated for each development stage and in each soil treatment, giving a total of 175 plants cultivated. We scrutinized potential changes in allometric relationships between root and shoot biomasses. Analysis of allometric relationships between root and shoot areas offered a complementary functional view of the equilibrium between resource capture surfaces. Several indicators of plant ability to capture resources (i.e. the amount of water transpired, leaf chlorophyll and nitrogen concentration, and number of root nodules) were also measured.

Research sample

The dwarf bean (Phaseolus vulgaris, cv. Oxinel, ® Vilmorin) was chosen as a model plant species because of its known plasticity of biomass allocation, both for wild and selected genotypes. In addition, it is a species commonly used as bio-assays in ecotoxicology due to its sensitivity to soil pollution. Seeds of similar weight [0.22; 0.30g] were selected to avoid large differences in seed reserves. After soaking for 4 h in tap water, three seeds were sown in each of the pots. Germination took 11 to 16.2 days depending on the soil treatment and this time increased with soil contamination. As a large majority of the seeds germinated, 1 seedling per pot was selected randomly and kept for the experiment.

Sampling strategy

In agreement with former studies regarding biomass partitioning, we performed standard major axis regression between dwarf bean shoot and root masses and areas. It enabled us to delineate biomass partitioning changes due to plant size modification (allometric effects) from other changes. Because we needed to ensure a wide range of plant size for such analysis, we cultivated and harvested dwarf beans at 5 development stages (from the end of cotyledon opening-stage 1 to 6 trifoliate leaves-stage 5). The development stages were not included in the analysis because we directly investigated the relationship between shoot and root masses or areas (and according to the different soil treatments). 5 replicates were used for each development stage, giving 25 dwarf bean plants cultivated, harvested and included in the standard major axis regression for each kind of soil. Rationally, 25 independent individuals are enough to perform (standard major axis) regression for a given soil, and to compare relationships between soils.

Data collection

During plant growth, at each watering (every each 2 to 3 days, see next answer) the mass of water added to maintain the pot at 60% of soil water holding capacity was recorded as the amount of water taken up and transpired since the last watering. The last 10 days before harvest were considered for calculation and analysis of plant transpiration. On the day of harvest, plant parts were separated (stem, leaves, and roots). Roots were washed gently with water and nodule numbers were counted. All organs were scanned and analysed to determine their area (software Winfolia for leaves and stems, WinRhizo for roots, Regents Instruments, Quebec, Canada). Then all plant samples were dried and weighed. The whole process determined the dry biomass of plant parts, their area, as well as Specific Leaf Area (SLA, cm2.g-1) and Specific Root Area (SRA, cm2.g-1). After drying and grinding (Retsch PM4 planetary grinder, Retsch, Haan, Germany), leaf N concentration was measured by an elemental analyser (NA 1500 NCS, Carlo Erba, Milan, Italy) for a subset of 112 samples encompassing all soil treatments and a wide range of plant size. Additonnaly, we measured chlorophyll a, b and other carotenoid concentrations in bean leaves. The day before harvesting, two 0.8 cm-diameter pieces of recently produced leaves were collected from the middle of each blade. They were placed in 3 mL of cooled (4°C) N-dimethylformamide (DMF) at 4°C for 48h. After extraction with DMF, Chl a, Chl b and total carotenoids in the extracts were measured spectrophotometrically. Data were collected by the corresponding author and one other co-author.

Timing and spatial scale

After soaking on March 21st 2016 pots were watered every 2 or 3 days and weighed to maintain the water holding capacity of soil at 60 %. First plants (first development stage) were harvested on April 14th, and last plants were harvested on June 14th.

Data exclusions

No data were excluded from the analysis.

Reproducibility

This study was conducted in a control environment (a greenhouse), with soils of known origin (same soils can be harvested and used again) and seeds of controlled origin (dwarf bean, Phaseolus vulgaris, cv. Oxinel, ® Vilmorin). All data were collected according to standardised protocols (For instance ISO 11274 for soil water holding capacity; NF ISO 10694 and NF ISO 11263 for soil C,N, P characterisation) and dedicated softwares were used (Winfolia and Winrhizo, Regent Instruments)

Randomization

Each plant was grown in a single pot and has a single identification number (IDs from 1 to 175). In the greenhouse, 175 locations (25 rows of 5 pots) were identified and numbered from 1 to 175. At the beginning of the experiment, random permutation of pot IDs provide the initial location of all pots (the first ID of the permutation result in the first location and so on). Then, new random permutation of pots ID was done each 15 days, and pots were moved accordingly. This enabled to avoid any spatial dependency between sample units and soil treatments.

Blinding	A single identification number was attributed to each pot and corresponding plant. During all data collection, this number was used without reference to the corresponding soil treatment. Plant harvesting (and following measurements) was based on objective criterion (the number of leaves). If we cannot exclude some uncertainty during plants handling, possible errors are independent of soil treatments so that data collection was not biased.

Did the study involve field work? ☐ Yes ☒ No

Reporting for specific materials, systems and methods

We require information from authors about some types of materials, experimental systems and methods used in many studies. Here, indicate whether each material, system or method listed is relevant to your study. If you are not sure if a list item applies to your research, read the appropriate section before selecting a response.

Materials & experimental systems

n/a	Involved in the study	
☒	☐	Antibodies
☒	☐	Eukaryotic cell lines
☒	☐	Palaeontology and archaeology
☒	☐	Animals and other organisms
☒	☐	Human research participants
☒	☐	Clinical data
☒	☐	Dual use research of concern

Methods

n/a	Involved in the study	
☒	☐	ChIP-seq
☒	☐	Flow cytometry
☒	☐	MRI-based neuroimaging
